# Age-related changes in diffuse optical tomography sensitivity profiles from childhood to adulthood

**DOI:** 10.1117/1.JBO.27.8.083004

**Published:** 2022-07-09

**Authors:** Xiaoxue Fu, John E. Richards

**Affiliations:** University of South Carolina, Department of Psychology, Columbia, South Carolina, United States

**Keywords:** diffuse tomography, near-infrared light spectroscopy, Monte Carlo simulation, head models, development

## Abstract

**Significance:**

Diffuse optical tomography (DOT) uses near-infrared light spectroscopy to measure changes in cerebral hemoglobin concentration. Anatomical interpretations of the brain location that generates the hemodynamic signal require accurate descriptions of the DOT sensitivity to the underlying cortex. DOT sensitivity profiles are different in infants compared with adults. However, the descriptions of DOT sensitivity profiles from early childhood to adulthood are lacking despite the continuous head and brain development.

**Aim:**

We aim to investigate age-related differences in DOT sensitivity profiles in individuals aged from 2 to 34 years with narrow age ranges of 0.5 or 1 year.

**Approach:**

We implemented existing photon migration simulation methods and computed source–detector channel DOT sensitivity using age-appropriate, realistic head models.

**Results:**

DOT sensitivity profiles change systematically as a function of source–detector separation distance for all age groups. Children displayed distinctive DOT sensitivity profiles compared to older individuals, and the differences were enhanced at larger separation distances.

**Conclusions:**

The findings have important implications for the design of source–detector placement and image reconstruction. Age-appropriate realistic head models should be used to provide anatomical guidance for standalone DOT data. Using age-inappropriate head models will have more negative impacts on estimation accuracy in younger children.

## 1 Introduction

Diffuse optical tomography (DOT) uses near-infrared light spectroscopy (NIRS) to indirectly measure neural activities by estimating changes in cerebral hemoglobin concentration.^1^^,^^2^ Conventional NIRS instruments provide multiple pairs of source and detector optodes that are placed on the scalp to form measurement channels that detect hemoglobin changes in the brain. High-density DOT (HD-DOT) instruments provide better spatial resolution using overlapping channels with multiple source–detector separation distances.^3^ For both types of instruments, characterizing DOT sensitivity profiles is important for determining the extent to which a given channel can detect hemoglobin changes in the underlying brain tissues and localizing the specific brain regions being sampled.^4^

It is difficult to experimentally examine the penetration of near-infrared light into a participant’s head.^4^ Instead, Monte Carlo simulations can be used to simulate the propagation of photons through head and brain tissues.^5^ The forward model that estimates DOT sensitivity can then guide DOT image reconstruction to recover the cortical locations of hemoglobin concentration changes.^6^^,^^7^ Accurate forward solutions depend on the use of realistic head models that account for age-related structural changes in the head.^8^^,^^9^ DOT sensitivity profiles have been studied in adults. However, existing evidence has indicated that the characteristics of DOT sensitivity profiles in infants aged between 2 weeks and 2 years were different than those in adults between 20 and 24 years.^10^ There are continuous changes in the anatomical structures of the brain and extracerebral tissues beyond infancy.^11^^,^^12^ This study aimed to examine age-related differences in DOT sensitivity profiles using age-appropriate, realistic head models with extensive coverage from early childhood (2 years) to adulthood (30 to 34 years).

### 1.1 Use of Monte Carlo Simulations to Estimate DOT Sensitivity Profile

Monte Carlo simulations can be used to estimate the sensitivity of the DOT signal to the hemoglobin changes inside the head. Monte Carlo simulations provide numerical solutions to the radiative transport equation that models the photon propagation through head tissues.^2^^,^^13^ Modern graphics processing units allow the Monte Carlo approach to be computationally efficient to provide accurate modeling of photon–tissue interaction.^14^^,^^15^ Boas et al.^5^ described details of the input parameters and procedures for Monte Carlo simulations. Research using simulations need to (1) segment the MRI(s) to define head tissue types; (2) assign optical properties of tissue types to each voxel; (3) specify injection point and the direction of the photons; and (4) apply an algorithm to describe the path of the photon through the tissues. Steps 3 and 4 are repeated to compute the accumulative photon fluence and the total path length traveled through the head tissues.^4^

DOT sensitivity can be estimated from Monte Carlo simulation outputs. The outputs are fluence distribution of photon travel across the head. DOT sensitivity indicates the extent to which the detected DOT signal is influenced by the optical properties of the given volume of sampled tissue.^16^ One representation of DOT sensitivity is the spatial sensitivity profile that depicts the fluence distribution of detected photons.^5^ One approach to calculate DOT sensitivity is the “photon measurement density function” (PMDF)^8^^,^^17^ or “3-point Green’s function”^4^ or “source-detector channel DOT” (S-D channel DOT).^10^^,^^18^^,^^19^ The S-D channel DOT represents the probability that a photon injected from the source and measured at the detector location has traveled through a given tissue. S-D channel DOT is quantified at each voxel location where a photon traverses through. “Sampling depth” represents the depth of the photon measured in the head medium.

### 1.2 DOT Sensitivity Profile by Source–Detector Separation Distances

The DOT sensitivity changes as a function of source–detector separation distance in both adult and child head models. With increased source–detector separation distances, the fluence distribution covers wider tissue regions, extends deeper into the brain, and decreases in DOT sensitivity. These characteristics of DOT sensitivity profiles have been found in adult,^4^^,^^16^^,^^20^^,^^21^ infant,^9^^,^^10^ and child head models.^10^ For example, studies using adult head models have found that with short separation distances (20 mm), the fluence distribution was confined to shallow layers of the head model.^4^^,^^20^ DOT sensitivity to the brain increased linearly from 20 to 45 mm separation distance.^4^^,^^16^^,^^20^^,^^21^ The increase became less steep at separation distances beyond 45 mm.^4^^,^^20^ Fu and Richards^10^ quantified S-D channel DOT profiles as S-D channel DOT fluence strength as a function of the sampling depth. They studied the profiles across a range of infant, child, and adult ages (2 weeks to 2 years with narrow age bands, 4 years, 12 years, and 20 to 24 years). The DOT sensitivity profile peaked earlier for shorter than for larger separation distances. However, the fluence value dissipated more quickly for shorter than for larger separation distances. The DOT sensitivity profiles showed a trade-off between penetration depth and signal intensity. The DOT sensitivity profile extended to deeper cortex at larger source–detector separation distances, but the fluence strength systematically reduced with increasing separation distances in head models from adult^4^ and developmental samples.^10^ The optimal separation distances for measuring cortical activities need to be determined based on the achievable signal strength for the measurement device, the DOT sensitivity, and the desired spatial resolution of the measurement regions.^2^^,^^4^^,^^10^

There are age-related differences in changes of DOT sensitivity profiles as a function of source–detector separation distance. Photon propagation through the head tissues is influenced by anatomical structures of the brain and extracerebral structures.^20^^,^^22^^,^^23^ Fu and Richards^10^ showed that older infants and toddlers (6 months to 2 years) had different sensitivity profiles compared to young infants (2 weeks to 4.5 months), children (4 and 12 years), and adults (20 to 24 years). These age-related differences increased with larger source–detector separation distance. The older infants and toddlers displayed sensitivity profiles characterized by smaller peaks and more gradual declines with increased sampling depth at all separation distances from 10 to 60 mm. In contrast, young infant groups showed sensitivity profiles with higher peaks and more rapid decline as photons traveled deeper into the tissues. The same proportion of fluence strength carried deeper into the head tissues for the young infants, children, and adults than for the older infants and toddlers across separation distances. Moreover, the between-channel variance of the S-D channel DOT fluence as a function of sampling depth increased with larger separation distances in young infant groups. The increase was the most observable in older infants and toddlers, whereas the between-channel was stable for the child and adult groups.

The DOT sensitivity profiles as a function of source–detector separation distance have not been charted between childhood and early adulthood, though there are reasons to believe they differ across these ages. There are nonlinear developmental changes in whole head volume, cortical gray matter (GM), and white matter (WM) volumes from childhood to adulthood.^12^^,^^24^ The whole head volume increases until early to mid-adolescence and remains relatively stable in later life.^24^ The GM volume increases during early development, reaches the highest volume in childhood, steadily declines from adolescence to early adulthood, and displays decelerating decline from the third decades of life.^12^^,^^24^ The WM volume increases until late adolescence and shows a gradual decline from adulthood.^12^^,^^24^ White et al. examined DOT sensitivity in head models of children between 5 and 11 years old^25^ and found that age was positively related to DOT sensitivity in the frontal and occipital regions. Increased scalp-to-brain distance was associated with reduced path length through brain regions in children.^25^ Greater extracerebral volumes were also related to reduced sensitivity to the GM across source–detector separation distances from 20 to 50 mm in adults.^20^ Hence, the age-related changes in head and brain anatomy and composition from early childhood to adulthood may affect DOT sensitivity profiles. However, existing studies used head models of limited age ranges in childhood (e.g., 5 to 11 years,^25^ 4 years, and 12 years^10^) and do not track age-related changes or stability in DOT sensitivity profiles from early childhood to adulthood when developmental changes of the head and brain persist.

### 1.3 Present Study

This study examined age-related differences in changes of DOT sensitivity profiles across source–detector separation distances. We extended Fu and Richards’s study^10^ by computing S-D channel sensitivity across the period of childhood (2 to 11 years), adolescence (12 to 17 years), and early adulthood (18 to 34 years). We performed MCX photon migration simulations^14^ to model photon propagation through the segmented tissues in the individual head MRIs. We examined S-D channel DOT fluence as a function of sampling depth. We also assessed the changes of the DOT sensitivity profiles from 10 to 60 mm source–detector channel separations. We expect to find age-independent characteristics of DOT sensitivity profiles, such as the relation between fluence values, sampling depth, and source–detector separation distance. We expect to find age-related differences in sensitivity profiles across separation distances between child and adolescent ages than adult ages. This study will supplement existing findings using infant head models^10^ to inform age-specific decisions of channel placements for NIRS recordings and the use of age-appropriate methods for making anatomical interpretations of stand-alone NIRS data across development.

## 2 Methods

### 2.1 Participants

The participants were 837 typically developing individuals ranging from 2 to 41 years of age. The MRIs were obtained from open-access databases, a local scanning facility, and collaboration study sites. Table 1 presents the number of MRIs obtained from the databases, separately for age and sex. The sample consisted of 132 participants from the Autism Brain Imaging Data Exchange (ABIDE),^26^ 8 from the Baby Connectome Project (BCP),^27^ 44 from the Child Mind Institute Healthy Brain Network (CMIHBN),^28^ 65 from the Infant Brain Imaging Study (IBIS),^29^ 57 from the Pediatric Imaging, Neurocognition, and Genetics Data Repository (PING),^30^ and 531 scans from data collected at the McCausland Center of Brain Imaging (MCBI) or drawn from collaborative studies at other sites. The age spans for groups were 1 year for most of the child and adolescent ages, and 0.5 years for some age groups with enough participants. The adult age groups covered a 5-year span. All studies had institutional review board approval and informed consent. The University of South Carolina Institutional Review Board approved data collection at the MCBI and the use of data from all open-access databases.

**Table 1:** Demographical information of study participants by age group, sex, and data source.

Participant information			Data source					
Age group (Years)	Total N	Mean age (SD)	ABIDE N	BCP N	CMIHBN N	IBIS N	MCBI and collaboration sites N	PING N
2	74	2.07(0.11)	0	8	0	65	1	0
3	36	3.40(0.31)	0	0	0	0	12	24
4	24	4.26(0.19)	0	0	0	0	10	14
5	62	5.23(0.14)	0	0	43	0	0	19
6	10	6.30(0.17)	0	0	0	0	10	0
7	16	7.55(0.29)	5	0	0	0	11	0
8	35	8.43(0.31)	22	0	0	0	13	0
9	19	9.63(0.26)	8	0	0	0	11	0
10	20	10.30(0.21)	6	0	0	0	14	0
11	2	11.44(0.23)	0	0	0	0	2	0
12	29	12.36(0.19)	9	0	0	0	20	0
12.5	21	12.79(0.26)	0	0	0	0	21	0
13	22	13.16(0.20)	11	0	0	0	11	0
13.5	33	13.74(0.14)	25	0	0	0	8	0
14	47	14.28(0.25)	21	0	1	0	25	0
15	11	15.55(0.40)	0	0	0	0	11	0
16	49	16.34(0.29)	2	0	0	0	47	0
17	15	17.31(0.25)	0	0	0	0	15	0
18	27	18.29(0.18)	9	0	0	0	18	0
18.5	28	18.71(0.14)	6	0	0	0	22	0
19	23	19.19(0.09)	4	0	0	0	19	0
19.5	24	19.72(0.13)	4	0	0	0	20	0
20 to 24	138	22.25(2.83)	0	0	0	0	138	0
25 to 29	39	26.54(1.72)	0	0	0	0	39	0
30 to 34	33	32.19(1.47)	0	0	0	0	33	0

Note: ABIDE, Autism Brain Imaging Data Exchange; BCP, Baby Connectome Project; CMIHBN, Child Mind Institute Healthy Brain Network; IBIS, Infant Brain Imaging Study; MCBI, McCausland Center for Brain Imaging; PING, Pediatric Imaging, Neurocognition, and Genetics Data Repository.

### 2.2 MRI Sequences

This study used T1-weighted (T1W) and T2-weighted (T2W) scans from each collection site. Details of the MRI acquisition protocols have been described in the literature on the Neurodevelopmental MRI Database.^31^^–^^36^ All MRIs were converted to NIFTI compressed format with 32-bit floating point resolution. Bias-field inhomogeneity correction (N4 algorithm) was performed on the extracted T1-weighted images.^37^^,^^38^

### 2.3 MRI Preprocessing and Segmentation

The brains were extracted from the whole-head MRI volume in a procedure adapted from the FSL VBM pipeline.^39^ The T1W volume for each participant was registered to an age-appropriate average MRI template. The MRI templates are an unbiased representation of the average for the age group. The average templates came from the MRI database.^34^^–^^36^ The brain from the average template was transformed into the participant MRI space and used as a mask on the head volume. The extracted masked data were then used with FSL brain extraction tool program.^40^^,^^41^ Each brain was visually inspected and manually modified if necessary.

**Fig. 1:**
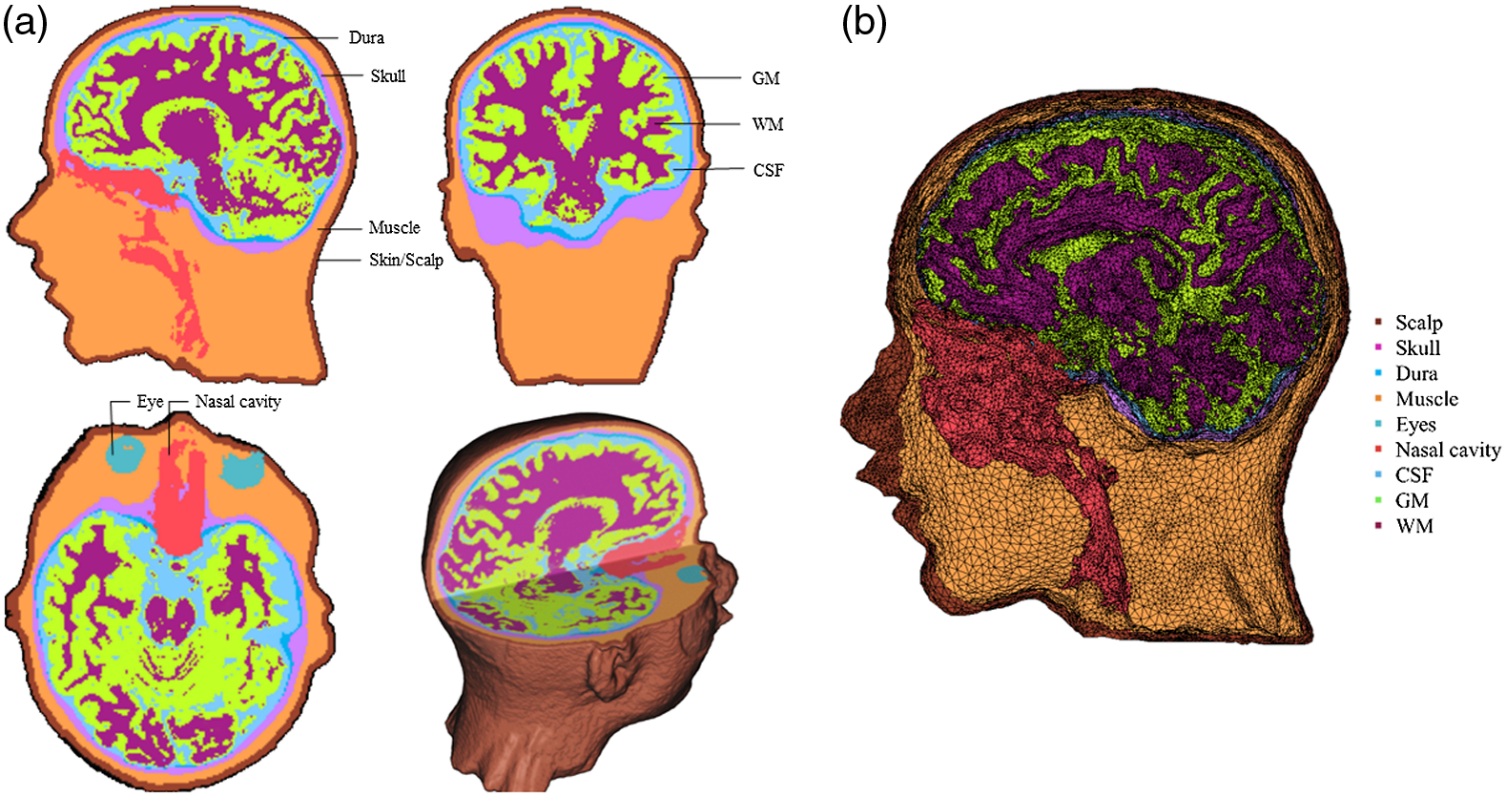
Segmented head MRI volumes for an adolescent MRI. (a) The segmented head model. (b) The segmented head model with dense FE mesh (segmented FE mesh).

We performed “finite element method” (FEM) segmentation. Each head MRI volume was segmented into nine media types: GM, WM, cerebrospinal fluid (CSF), other brain matter, skull, scalp, eyes, air, and other inside skull materials. Details of the segmentation methods are presented in the Supplemental Material. The segmented regions were assembled into a single MRI volume that we will refer to as the “segmented head MRI volume.” Figure 1(a) shows a three-dimensional rendering of the T1W volume from a 12-year-old adolescent with a cutout revealing the segmented MRI volume. The realistic head model represents the geometry of the head and allows us to differentiate optical properties of different tissue types.

### 2.4 Mesh Generation

A “finite element” (FE) tetrahedral mesh was constructed for each segmented head MRI volume. Figure 1(b) displayed the dense meshes that were produced using the iso2mesh toolbox with CGAL 3.6 mesh program (“v2m” function^42^). Tetrahedral meshes accurately represent the boundaries of complex 3D volumetric tissues and increase the accuracy in modeling photon propagation in complex mediums such as the head and brain.^43^ The FE volumetric meshes have nodes that represent the voxel locations for the tetrahedra, a four-element matrix representing the corners of each tetrahedron, and a vector representing the media type from the segmented head MRI volume. The dense “segmented FE mesh” was used for MCX^14^ to find a segment element that was closest to an electrode position.

### 2.5 Scalp Location

#### 2.5.1 Virtual optodes placement

**Fig. 2:**
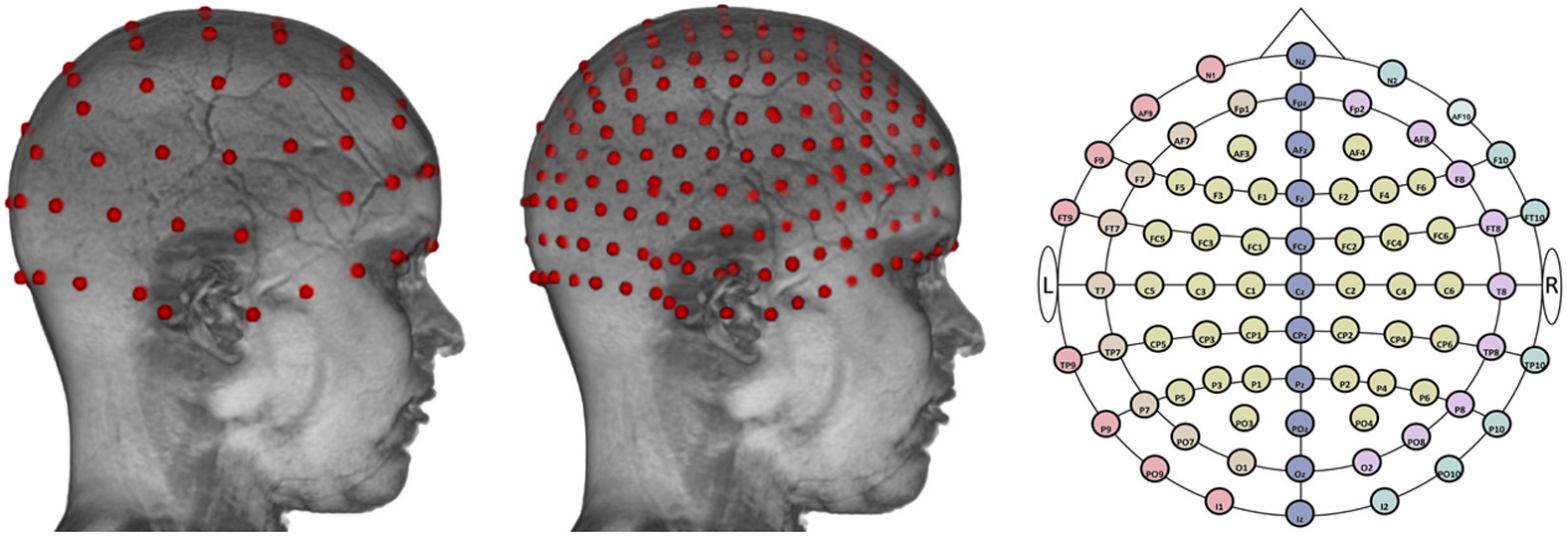
Virtual optodes placement. From left to right: a 12-year-old individual head model with virtual optodes placed on the 10-10 electrode system, the same 12-year-old individual head model with virtual optodes placed on the standard 10-5 electrode system, and a two-dimensional layout of the 10-10 system).

The locations for the 10-10 and 10-5 electrode systems^44^ were constructed on each head MRI volume. These standard locations were used for virtual optode placement. We used “electrode(s)” when referring to the 10-10 or 10-5 electrode systems. “Optode(s)” and “channel(s)” were used when referring to the NIRS sensors and the positioning of these sensors. We first manually marked cranial fiducial points using MRIcron:^45^^,^^46^ nasion (Nz), vertex (Vz), inion (Iz), left preauricular point (LPA), right preauricular point (RPA), left mastoid (LMa), and right mastoid (RMa).^47^ The coordinates of the fiducials were transferred onto the scalp mesh. This was followed by calculating 81 virtual positions based on the “unambiguously illustrated 10-10 system.”^44^ Details for constructing the 10-10 locations are described by Richards et al.^47^ and the Supplemental Material. We simulated a total of 358 optodes on 10-5 locations by calculating center points between 10-10 positions. Figure 2 shows the virtual optode placements at 10-10 and 10-5 electrode positions.

#### 2.5.2 Source–detector channels, all optode pairs

Source–detector channels were defined in two ways for the analyses. Source–detector channels: source–detector channel locations were defined using optode combinations centered on each 10-10 electrode location. The 10-10 electrode locations were centered between surrounding adjacent pairs of 10-10 or 10-5 electrode locations. The channel locations were used to estimate the “S-D channel DOT fluence” described below. All optode pairs: Some channel locations were defined from the combination of all 10-5 electrode positions for the source–detector separation distance analyses. The path from each 10-5 electrode location to all other 10-5 electrode locations was traced along the scalp and the separation distance of the path was recorded. The 10-5 electrode combinations formed a total of 63,903 paths. The channel location was defined at the half-way point in this path. The optode pairs, scalp path distances, and channel locations were recorded. Source–detector separation distances were calculated as half of the scalp path distances. The separation distances were used for the S-D channel DOT sensitivity profile analysis described as follows.

### 2.6 Photon Migration Simulations

Photon transport simulations were performed to estimate the DOT fluence values. Photon migration through the head and brain tissues for each FE segmented MRI volume was modeled using the Monte Carlo eXtreme package (MCX).^14^^,^^15^ The MCX uses a GPU-accelerated, voxel-based Monte Carlo simulation algorithm. The default voxel size $1\times 1\times {1\;\mathrm{mm}}^{3}$ was used. We launched $10^{8}\text{ photons}$ from the 358 10-5 positions for the time window of 0 to 5 ns. The wavelength was set at 690 nm. The optical properties of the nine tissue types were set in accordance with previous studies.^10^^,^^18^^,^^19^ Table 2 presents the input optical property values of the head media and the sources of these values (see also Ref. 10). The values of optical properties vary across studies. Fu and Richards (2021)’s Supplemental Table 1 summarized the optical properties used in adult and infant-child studies.^10^

**Table 2:** Optical properties used in Monte Carlo photon migration simulations. The wavelength was set at 690 nm. $\mu_{a}$ is the optical absorption coefficient, $\mu_{s}$ is the scattering coefficient, g is the anisotropy coefficient, and N is the index of refraction.

Tissue type	$\mu_{a}$ (${\mathrm{mm}}^{-1}$)	$\mu_{s}$ (${\mathrm{mm}}^{-1}$)	g	N
WM	0.07	40.1	0.85	1.37
GM	0.02	8.4	0.90	1.37
CSF	0.0004	1	0.99	1.37
Dura	0.0101	80	0.99	1.37
Skull	0.0101	100	0.99	1.37
Skin	0.0101	80	0.99	1.37
Muscle	0.0101	80	0.99	1.37
Eyes	0.0004	1	0.99	1.37
Nasal cavity	0.0101	80	0.99	1.37
Nonmyelinated axons	0.07	40.1	0.85	1.37

The Monte Carlo simulations estimate fluence distribution.^4^^,^^5^ The output of the simulations included the sum photon flux (or “fluence”) for each voxel. The number ranged from zero (i.e., all photons exited the media) to the total number of injected photons (i.e., all photons were absorbed). We took the log (fluence) as our number representing the DOT sensitivity for each voxel.

### 2.7 DOT Sensitivity Analyses

**Fig. 3:**
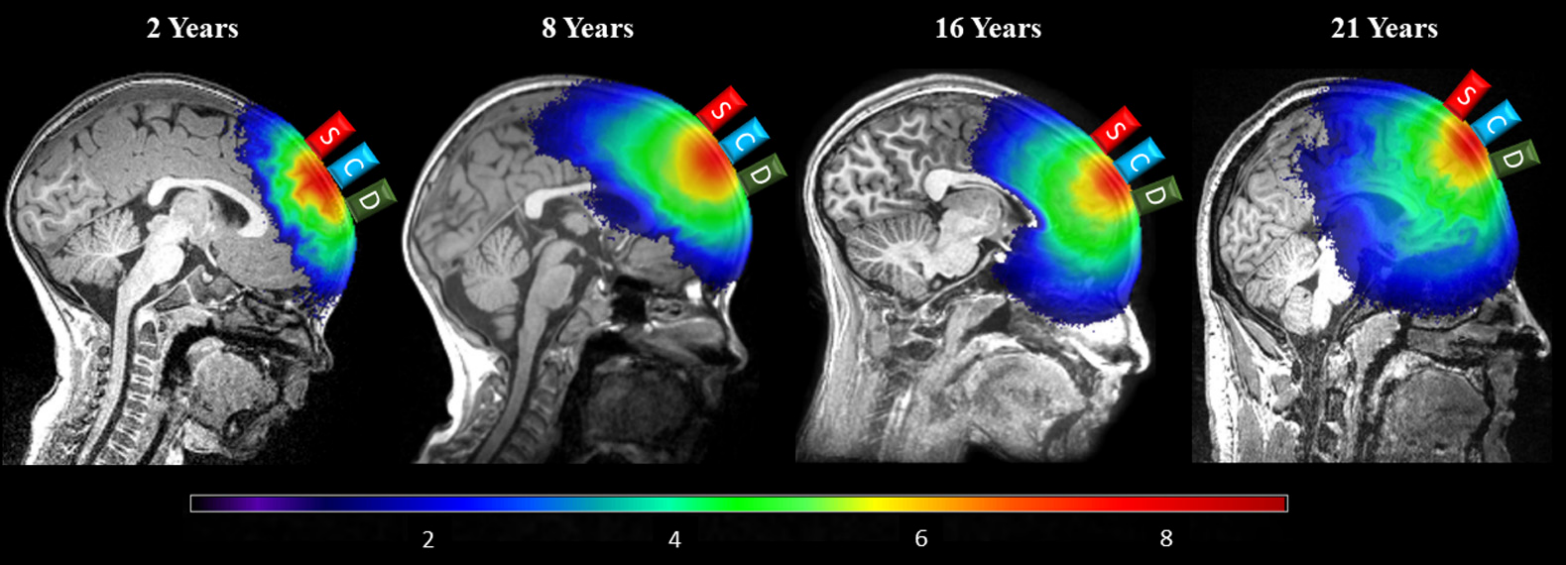
S-D channel DOT fluence distribution in example individuals in early childhood (2 years), late childhood (8 years), adolescence (16 years), and adulthood (21 years). Monte Carlo photon migration simulations were used to estimate fluence distributions. The red area represents greater fluence. Photons were launched from 10-5 locations. The channel location was defined as the half-way point in the path from a 10-5 location to another 10-5 location.

The output from the Monte Carlo simulations was used to compute the sensitivity for each source–detector channel. We calculated the voxel-wise “S-D channel DOT fluence” by multiplying the source-optode fluence distribution by the detector-optode fluence distribution.^10^^,^^18^ The S-D channel DOT fluence is a unitless measure that represents the sensitivity of the DOT measure for detecting changes in optical properties at a given point inside the head medium (PMDF;^8^^,^^17^ or “3-point Green’s function^4^). Figure 3 shows the S-D channel DOT fluence displayed on example MRIs for a single source–detector channel. This figure shows that the fluence distribution shows the expected characteristic across ages; there was a monotonic decrease in fluence strength as photons traveled deeper into the head tissues.

### 2.8 S-D Channel DOT Sensitivity Profile by Source–Detector Separation Distances

We examined changes in S-D channel DOT sensitivity profiles as a function of source–detector separation distance. At each voxel, we recorded the S-D channel DOT fluence value and the distance (depth) from the scalp channel location to the voxel with the fluence estimation. We refer to the distance from the channel to the location of the S-D channel DOT fluence value inside the head model as the “sampling depth.” Figure S1 (right panel) in the Supplemental Material provides a conceptual illustration of sampling depth. The S-D channel DOT sensitivity profile was defined as the fluence value at the voxel as a function of the sampling depth. The target source–detector separation distances for testing were from 10 to 60 mm by 5 mm increments. We set the minimum and maximum separation distances for each participant and each target separation distance to ensure about 500 channels are being sampled for the target separation. As it is not possible to have the number of channels at the exact target separation distance, we set a range of separation distances to sample the 500 channels, with the target separation distance as the median. For example, for a 12-year-old, there were 501 channels sampled at the minimum separation of 28.8 mm and maximum separation of 31.2 mm for the target separation of 30 mm. Figure S1 (left panel) in the Supplemental Material provides a visualization of the source–detector channels for the example participant. It shows that our channel selection ensures a good coverage of the head. Table S1 in the Supplemental Material lists the mean minimum and maximum separation distances for each target separation distance for each age group. For each participant and each channel with the target separation distance (e.g., 500 channels with 20 mm separation distance), we computed the S-D channel DOT fluence value at each sampling depth. The fluence values were then averaged across channels by sampling depth (1 to 100), target separation distance, and participant. We additionally examined the variance of the sensitivity profile by computing the standard error of the mean fluence value as a function of sampling depth. The shape of the S-D channel DOT fluence distribution was quantified by the half-width half-maximum (HWHM).^10^^,^^48^ The HWHM identified the sampling depth at which the fluence first dropped below half of the maximum value. We referred to this as the HWHM location.

## 3 Results

### 3.1 S-D Channel DOT Sensitivity Profiles by Source–Detector Separation Distances

**Fig. 4:**
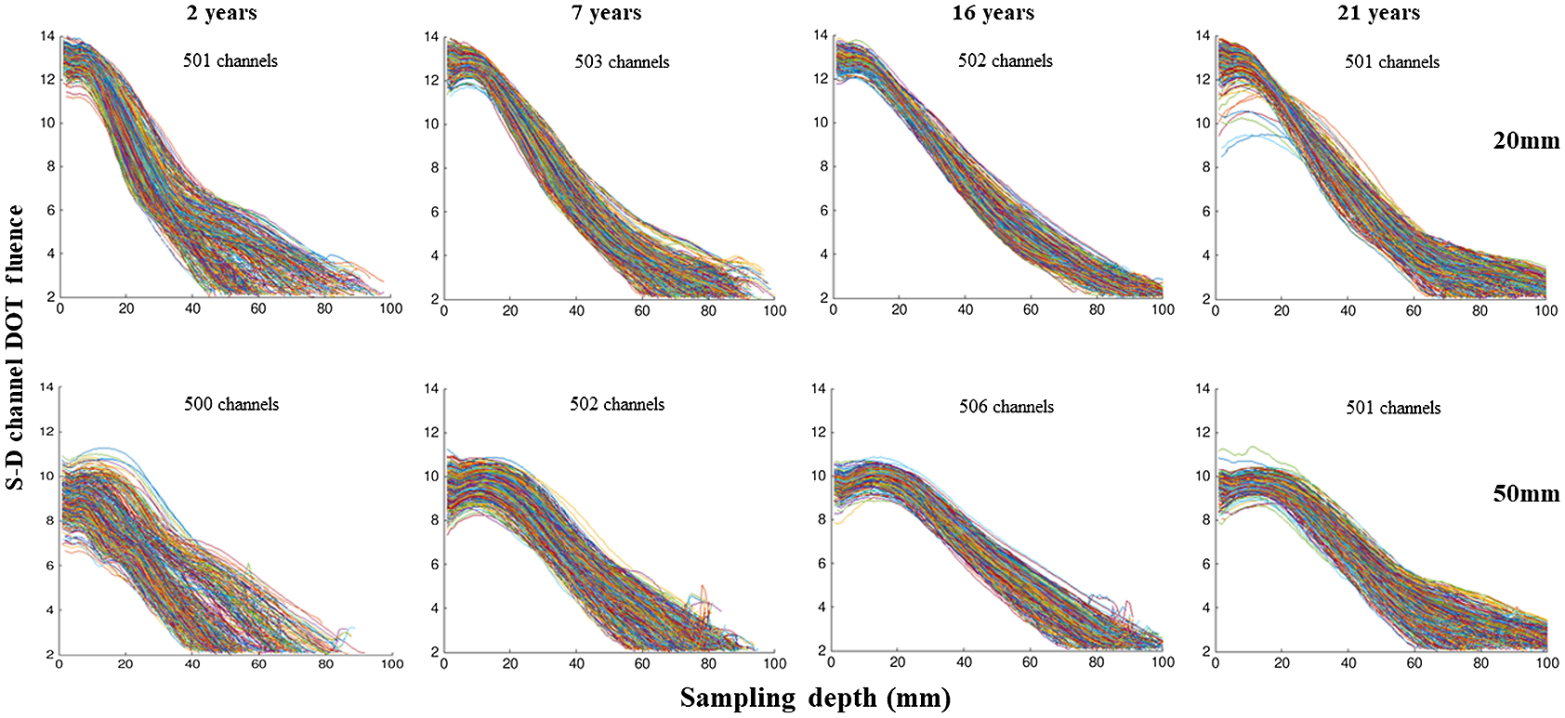
S-D channel DOT fluence sensitivity profile by channels. Four example individuals were selected, one from each age category (early childhood, late childhood, adolescence, and adulthood). For each participant and each channel with the target separation distance, we computed the S-D channel DOT fluence and the distance from the channel location to the voxel with the fluence estimation (sampling depth). The S-D channel DOT fluence value was plotted as a function of the sampling depth separately for channels at the source–detector separation distances for 20 and 50 mm for everyone. The number of channels that were sampled for each separation distance is provided for each individual figure.

The sensitivity profile at each target separation distance represents the S-D channel DOT fluence as a function of the depth between channel location and the voxel sampled in the fluence distribution (sampling depth). This was done by selecting optode pairs at 10-5 locations with separation distances from 10 to 60 mm by 5 mm increments and calculating the S-D channel DOT fluence for each pair and averaging over pairs of the same separation distance. Figure 4 presents examples of the S-D channel DOT sensitivity profiles plotted by channels (separate lines) at 20 and 50 mm separation distances (different panels) for one participant each at age 2, 7, 16, and 21 years. Additional examples are displayed in Fig. S2 in the Supplemental Material. The sensitivity profiles across the four individuals showed greater peaks and steeper slopes for channels at 20 mm than 50 mm separation distance. The examples show that there was between-channel variance across ages and separation distances. However, the between-channel variance at both separation distances was the greatest at age 2 and was reduced at older ages. The between-channel variance increased from 20 to 50 mm separation distances for the two child groups whereas it remained stable for the adolescent and adult groups. More systematic examinations of the group-level between-channel variance of the S-D channel DOT fluence as a function of sampling depth are presented in Sec. 3.2.

**Fig. 5:**
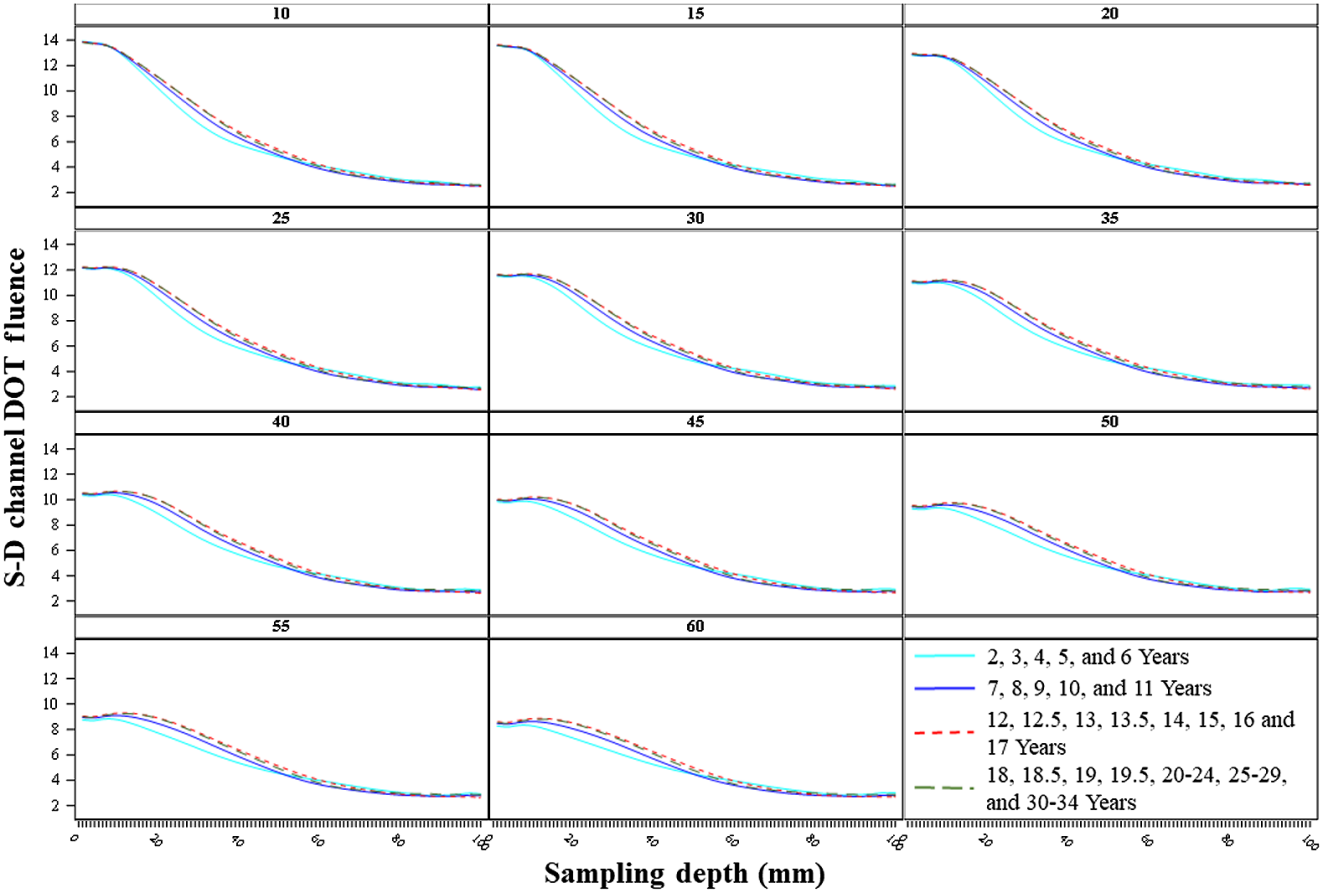
S-D channel DOT fluence sensitivity profile by age bins. The S-D channel DOT fluence value was plotted as a function of the sampling depth, defined as the distance (depth) from the scalp channel location to the voxel location in the head model where the S-D channel DOT fluence was measured. For visualization, we created four age bins: early childhood (2 to 6 years), late childhood (7 to 11 years), adolescence (12 to 17 years), and adulthood (18 to 30 to 34 years). Mean fluence values by each age bin are displayed.

The sensitivity profiles changed with separation distances across age groups. Figure 5 displays the S-D channel DOT fluence as a function of sampling depth separately for each target separation distance, averaged across channel locations, and separately for the four age bins. We grouped the samples into four age bins based on preliminary data visualization: early childhood (2 to 6 years), late childhood (7 to 11 years), adolescence (12 to 17 years), and adulthood (18 to 30 to 34 years). The sensitivity profiles changed systematically with separation distance. The peak of the sensitivity function was largest at 10 and 15 mm separation distances and decreased as the source–detector separation distance increased. The S-D channel DOT fluence values dissipated more quickly as photons traveled deeper into the tissues at shorter than longer separation distances. The sensitivity profiles became flatter with increasing separation distances. Compared with the sensitivity profile at shorter separation distances, the same fluence value was carried to greater sampling depth at larger separation distances.

**Fig. 6:**
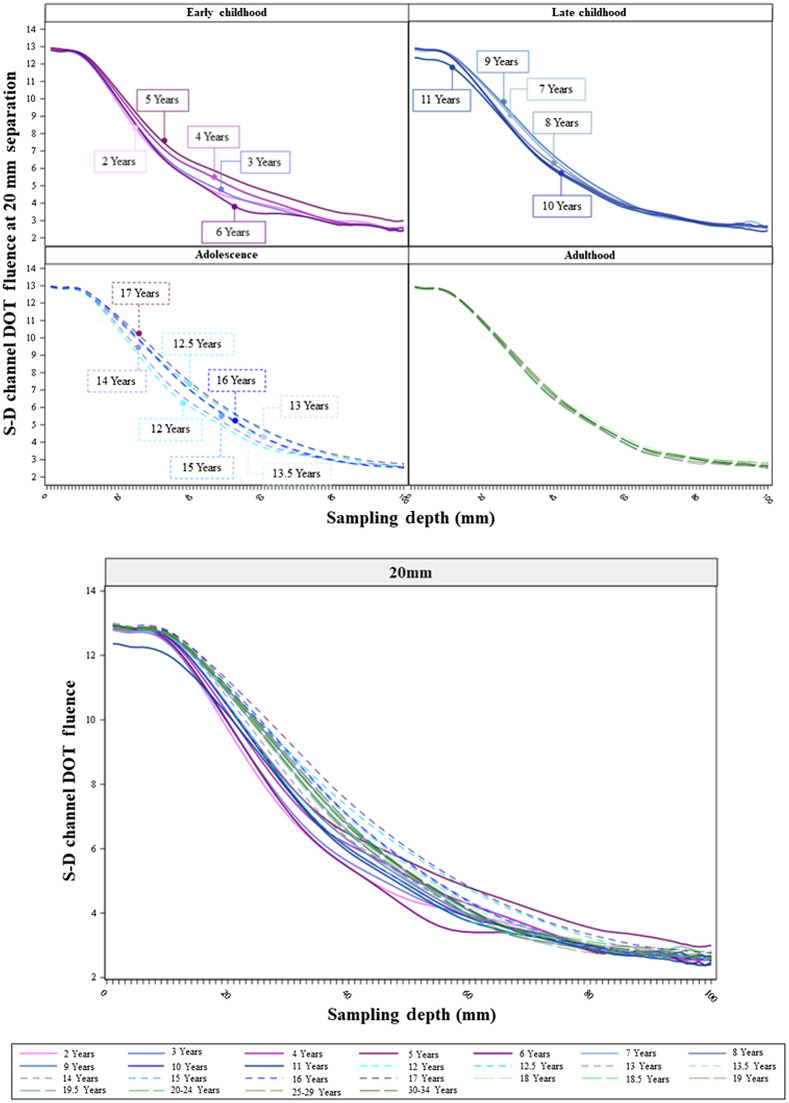
S-D channel DOT fluence sensitivity profiles by individual age groups at 20 mm source–detector separation distances. The upper panel presents the sensitivity profiles by age groups in age bins (as separate panels). The lower panel plots sensitivity profiles by age groups in the same panel.

**Fig. 7:**
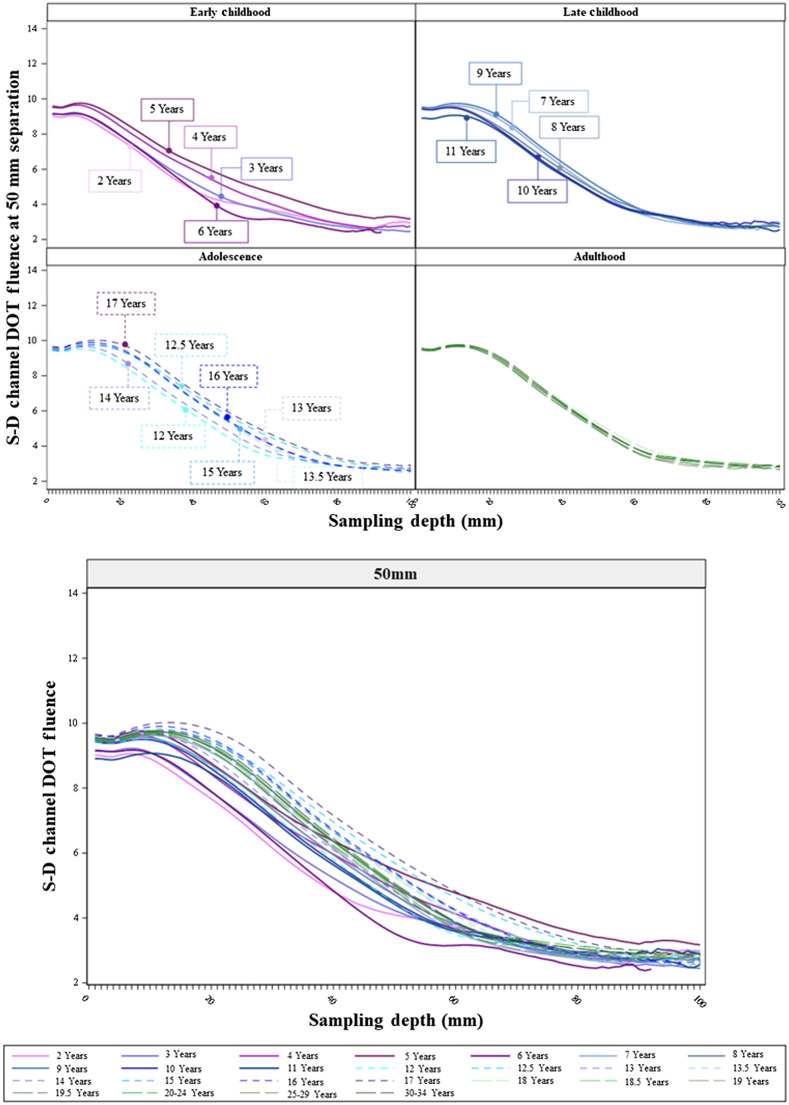
S-D channel DOT fluence sensitivity profiles by individual age groups at 30 mm source–detector separation distances. The upper panel presents the sensitivity profiles by age groups in age bins (as separate panels). The lower panel plots sensitivity profiles by age groups in the same panel.

**Fig. 8:**
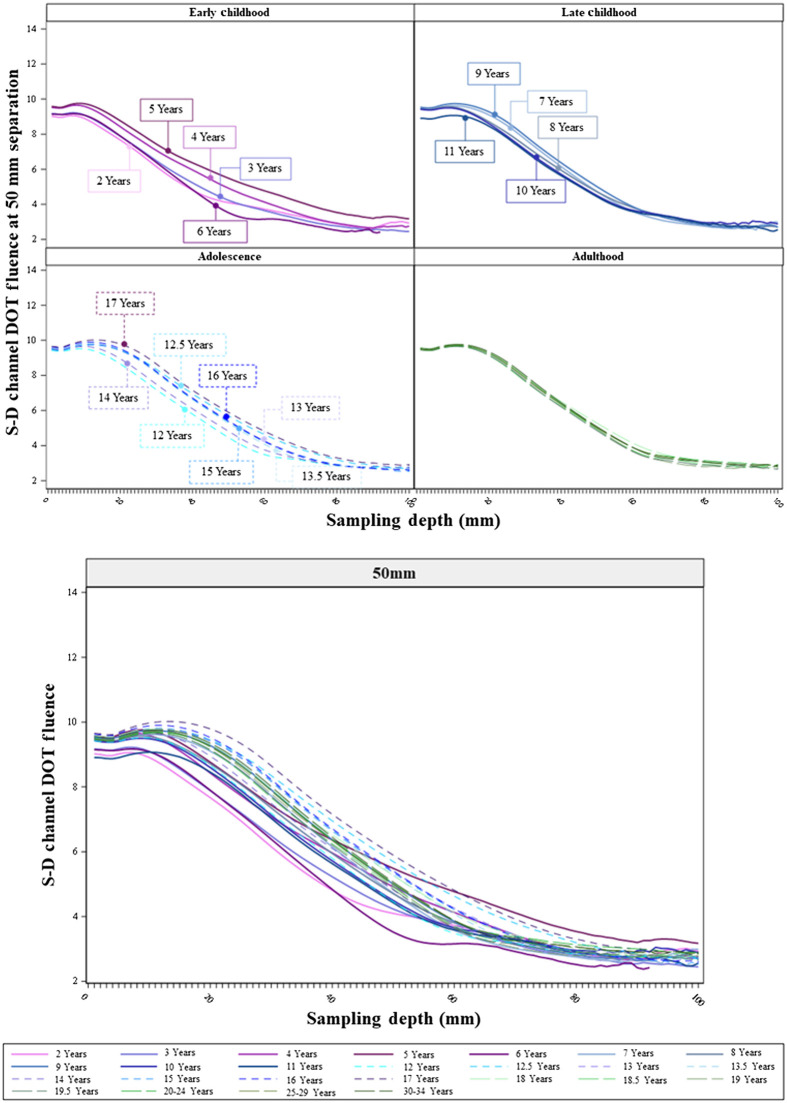
S-D channel DOT fluence sensitivity profiles by individual age groups at 50 mm source–detector separation distances. The upper panel presents the sensitivity profiles by age groups in age bins (as separate panels). The lower panel plots sensitivity profiles by age groups in the same panel.

The age-related differences in the sensitivity profiles showed consistent patterns across separation distances. Figures 6–8 show the S-D channel DOT sensitivity profiles for the 20, 30, and 50 mm source–detector separation distances, respectively, and separately for individual age groups. The patterns of age-related differences in the shape of the sensitivity profiles were consistent across separation distances. The age-related differences are most visible at the 50 mm separation distance (Fig. 8). The between-age-group differences were the greatest in the early childhood groups and declined from childhood to adulthood. The between-age-group differences in the early childhood groups were enhanced with increasing separation distances from 20 to 50 mm. The separation-related change was less visible in late childhood and adolescence groups. The between age-group differences in the adulthood groups remained minimal across separation distances. Consistent with Fig. 5, the early childhood groups had lower peaks than older age groups. However, the early childhood groups showed a less rapid decline in S-D channel DOT fluence values with increasing sampling depth (i.e., flatter shape) than the adolescence and adulthood groups. The shapes of the sensitivity profiles were similar between the adolescence and adulthood groups. Figures S3(a)–S3(c) in the Supplemental Material present the S-D channel DOT sensitivity profiles for the 20, 30, and 50 mm separation distances using participants’ age as a continuous variable. The visualizations further illustrate that the between-age variance was larger among children than adults. At larger separation distances (e.g., 50 mm), the adolescents and adults had S-D channel DOT fluence values that peaked at a larger sampling depth. However, the values declined more rapidly in adolescents and adults than younger ages.

#### 3.1.1 Summary

The findings revealed that the S-D channel DOT sensitivity profiles display common characteristics and show similar changes with increasing separation distances across age groups but also important age-related differences. Consistent with Fu and Richards’s findings,^10^ the S-D channel DOT fluence decreased exponentially in all ages as the photons traveled deeper into the head tissues. As source–detector separation distances increased, the peak of the sensitivity profiles was reduced and the decline in the fluence values became more gradual. A similar observation is that the early childhood groups (e.g., 2 years) had a flatter sensitivity profile than the adulthood groups (e.g., 20 to 24 years) at larger separation distances. We extended the previous findings by showing that the between-age differences are greater in childhood (2 years to 11 years) than adolescence (12 years to 17 years) and adulthood groups (18 to 34 years). The early childhood groups (2 to 6 years) displayed different sensitivity profiles than the adolescence and adulthood groups.

### 3.2 Variance of S-D Channel DOT Sensitivity Profiles

**Fig. 9:**
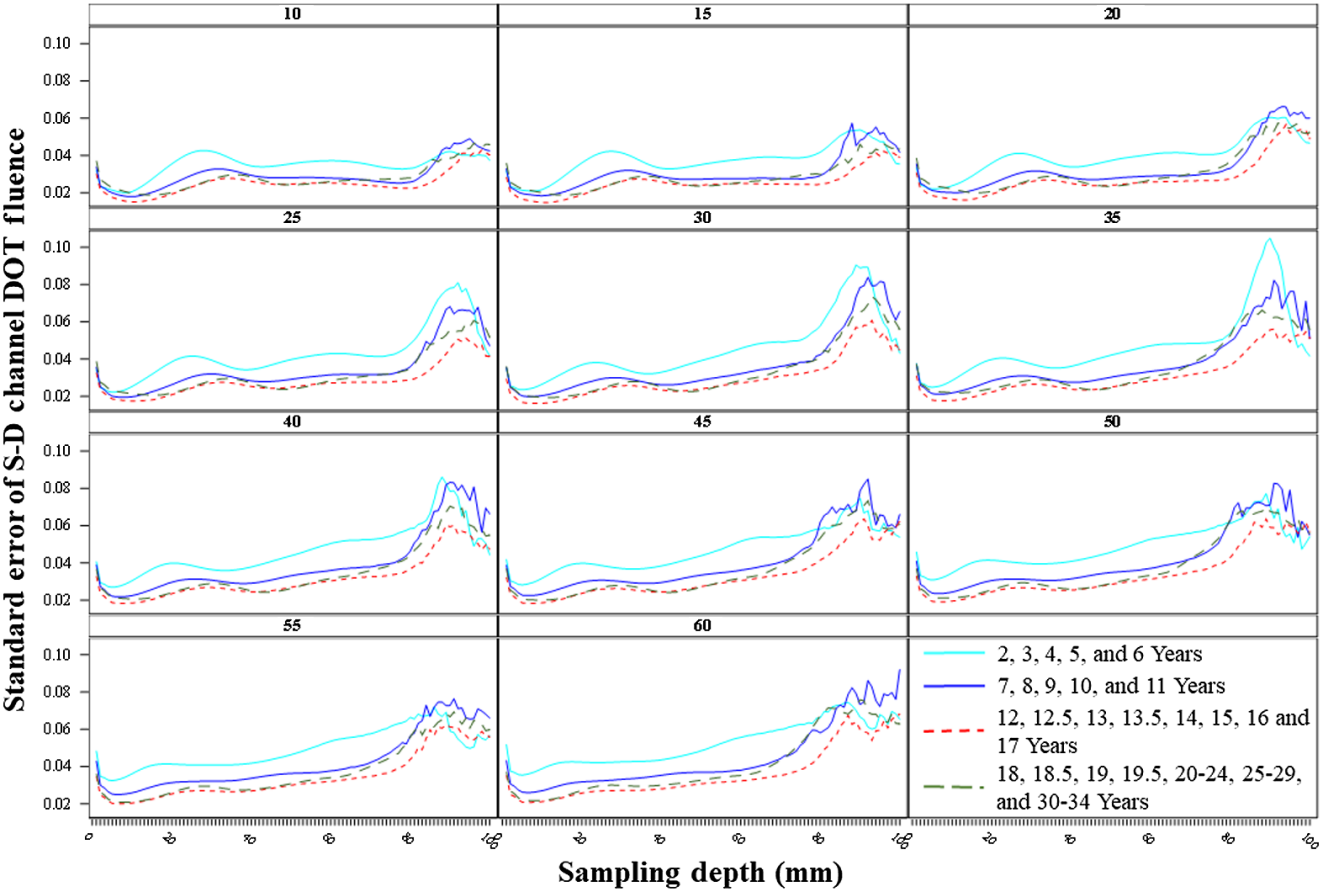
Variance of S-D channel DOT fluence sensitivity profile averaged by the age bins. This is the standard error of the S-D channel DOT fluence value as a function of the sampling depth.

**Fig. 10:**
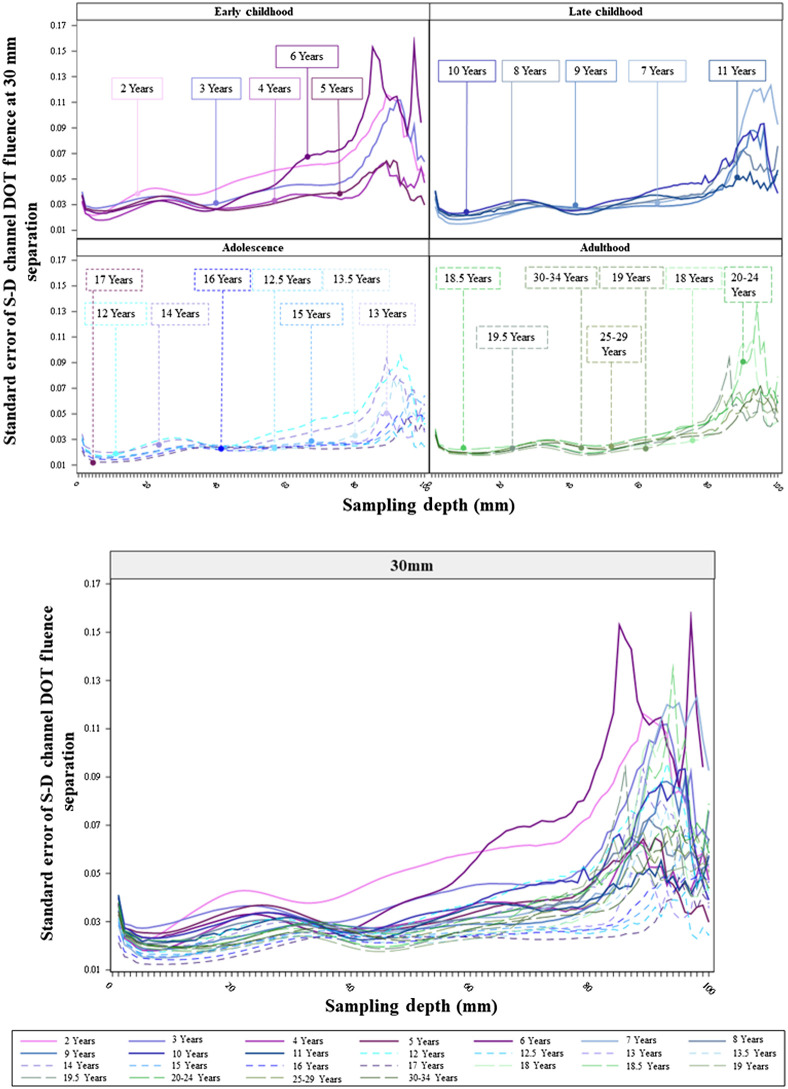
Standard error of the S-D channel DOT fluence sensitivity profile by individual age groups at 30 mm source–detector separation distance. The upper panel presents the variance as a function of sampling depth by age groups in age bins (as separate panels). The lower panel plots the variance by age groups in the same panel.

We examined the between-channel variance of the S-D channel DOT fluence as a function of sampling depth. Figure 4 indicates age-related differences in the between-channel variance of the sensitivity profiles in short and long separation distances. There was a larger increase in the between-channel variance with increased separation distance in the child groups than adolescent and adult groups. We further examined the variance by computing the average standard error of the mean S-D channel DOT fluence value. Figure 9 presents the average of the standard error as a function of sampling depth by age bins for each target separation distance. As expected, the average variance was greater in the early childhood groups than older age groups across sampling depth less than 80 mm. The average variance was the lowest in the adolescence groups. There was an increase in variance as a function of sampling depth with separation distances in the early childhood group, whereas the variance remained stable across separation distances for older age groups. All age bins displayed increasing average variance with increased sampling depth across separation distances. Figure 10 shows the standard error of the mean S-D channel DOT fluence value as a function of sampling depth separately by the individual age groups at 30 mm separation distance. We present similar figures for 20 and 50 mm separation distances in Figs. S4(a) and S4(b) in the Supplemental Material. The patterns between-age differences in the average variance as a function of sampling depth remained consistent across separation distances. The early childhood age groups (specifically age 2, 3, and 6 years) had larger variance across sampling depth and separation distances. The between-age-group difference in variance among early childhood groups was greater than late childhood, adolescence, and adulthood groups at sampling depth <80  mm across separation distances.

#### 3.2.1 Summary

The patterns of age-related differences in average variance as a function of sampling depth were consistent across separation distances. A consistent finding with Fu and Richards’s^10^ was that the early childhood groups showed increased variance across sampling depth with increasing separation distances. The variance as a function of sampling depth was stable across separation distances for late childhood, adolescence, and adult age groups. The between-age-group differences in variance were larger among early childhood groups than older ages.

### 3.3 Shape of S-D Channel DOT Sensitivity Profiles

**Fig. 11:**
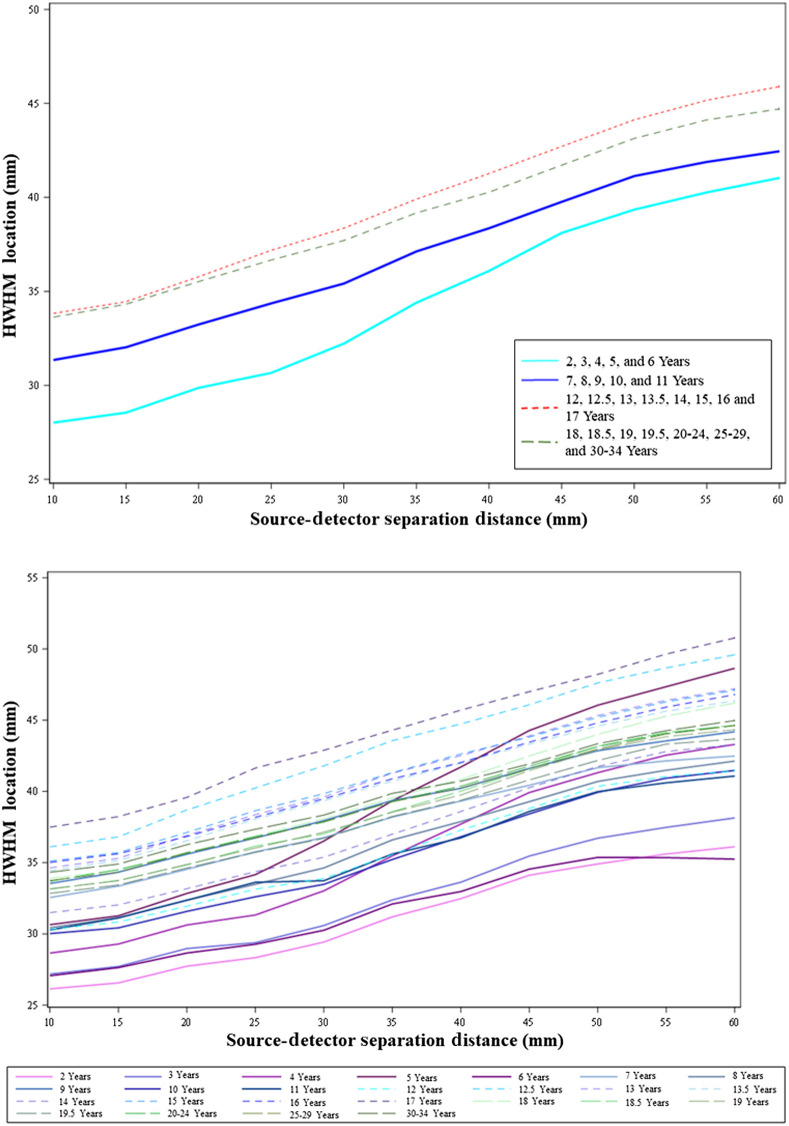
HWHM locations of S-D channel fluence as a function of source–detector separation distances. The figure shows age-related differences in how the shape of the S-D channel DOT sensitivity profile changes with separation distances. The HWHM location was defined as the point in the fluence distribution at which the fluence first dropped below half of the maximum value. The upper panel shows the mean HWHM locations as the function of source–detector separation distances separately for the four age bins: early childhood, late childhood, adolescence, and adulthood. The lower panel displays HWHM locations as the function of source–detector separation distances separately by individual age groups.

The last analysis examined the HWHM location for S-D channel DOT profiles. Figure 11 displays the HWHM location as a function of the source–detector separation distance separately for the four age bins (upper panel) and for individual age groups (lower panel). The HWHM location became deeper as the separation distance increased for all age groups. This increase indicates the apparent change in steepness in the S-D channel DOT fluence sensitivity profiles over separation distances (Fig. 5). The increasing trend in HWHM locations implies that there was an increase in sensitivity to deeper cortex when increasing the source–detector separation distances, though the fluence strength systematically decreased with increasing separation distance (e.g., Fig. 5). The patterns of age-related differences in HWHM locations over source–detector separation distances parallel those found in the sensitivity profiles (e.g., Fig. 5). Figure 11 (upper panel) shows that the mean HWHM locations across all separation distances were greater for the adolescence and adulthood groups and at the lowest levels for the early childhood group. The larger HWHM location values imply that the same proportion of fluence strength was carried to deeper cortical areas for adolescents and adults than for younger children. Figure 11 (lower panel) indicates that the between-age-group differences among early childhood groups increased at separation distances greater than 30 mm, and the between-age-group variance was greater than older age groups. This means that increasing source–detector separation distances has different effects on the fluence distributions across age groups between 2 and 6 years.

**Fig. 12:**
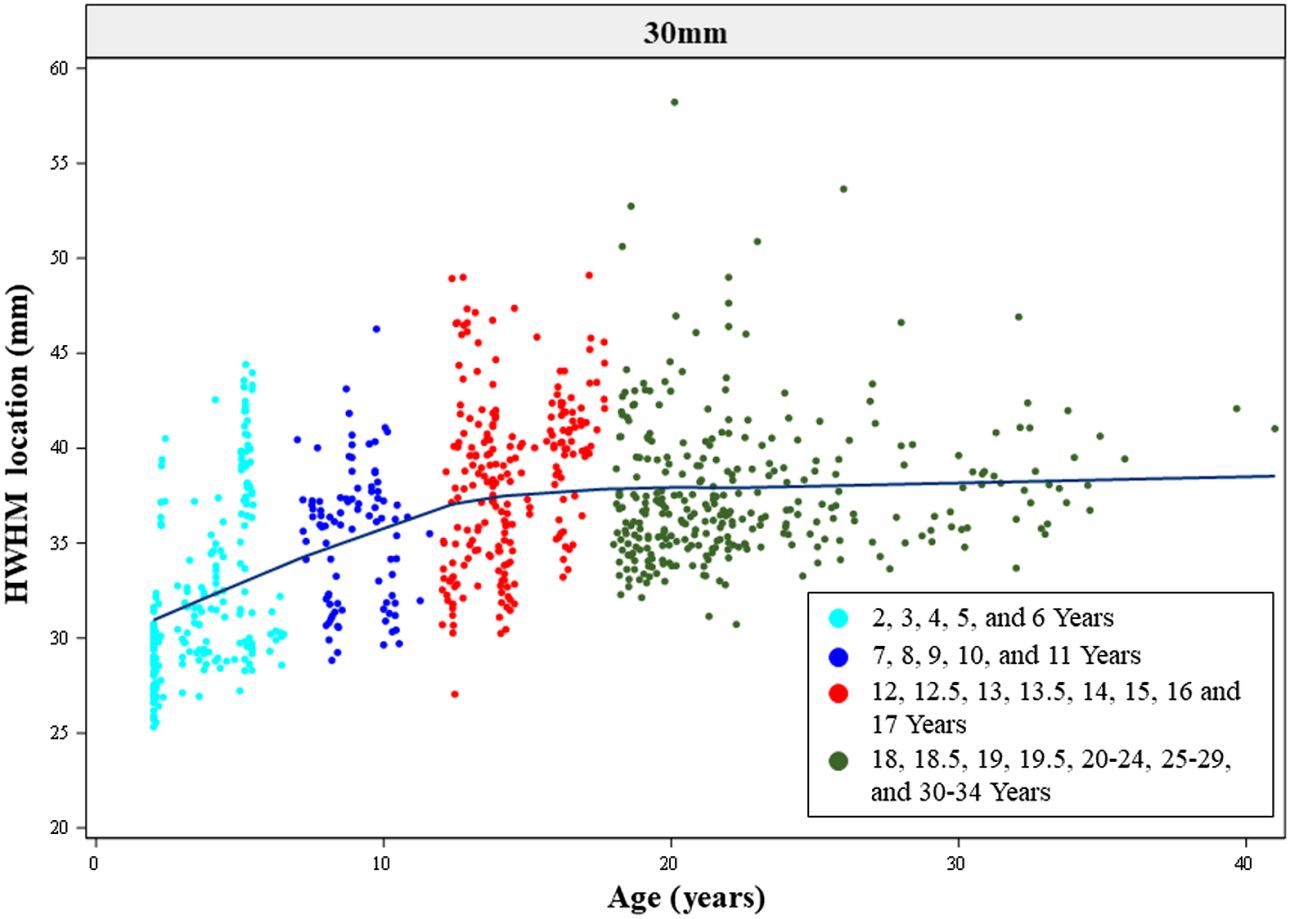
HWHM locations of S-D channel fluence as a function of age. The ages of individual participants were used as a continuous variable. The four age bins (early childhood, late childhood, adolescence, and adulthood) were color-coded.

We further explored changes in HWHM locations with age as a continuous variable by separation distances. Figure 12 and Figs. S5(a) and S5(b) in the Supplemental Material display the HWHM locations as a function of age at 30, 20, and 50 mm, respectively. They confirmed that the HWHM location values showed an increasing trend from early childhood to late childhood and to adolescence, and the values for adolescents and adults were comparable. The between-subject variance in HWHM locations among children between 2 and 6 years (early childhood) increased with increasing source–detector separation distances.

#### 3.3.1 Summary

The shape of S-D channel DOT sensitivity profiles showed a common characteristic across age groups, as found in Ref. 10. That is, the fluence distribution extends deeper into the head tissues as the source–detector separation distances increase. The important age-related difference was the same proportion of fluence strength carried deeper into the head tissues for adolescents, adults, and older children than younger children across separation distances. The between-individual variance in HWHM location values increased with larger separation distances for younger children.

## 4 Discussion

This study examined age differences in DOT sensitivity profiles as a function of source–detector separation distance. We implemented existing photon migration simulation methods using realistic head models in individuals ranging from 2 to 34 years with narrow age ranges of 0.5 or 1 year. We computed the S-D channel DOT that represents the probability that a photon has traveled through the brain tissue underlying the source and detector location. The sensitivity profiles displayed common characteristics across age groups. The same proportion of fluence strength extended deeper into the cortex at larger separation distances but showed lower peak fluence values than at shorter separation distances. The early childhood groups (2 to 6 years) displayed flatter sensitivity profiles than older age groups at larger separation distances (e.g., 50 mm). This means that the same proportion of fluence strength reached shallower tissues in early childhood than older age groups. The shape of sensitivity profiles in adolescence and adulthood groups was similar. The childhood groups (2 to 11 years) had greater between-age differences in both averaged sensitivity profiles across channels and between-channel variance of sensitivity profiles than adolescence and adulthood groups.

### 4.1 S-D Channel DOT Sensitivity Profile by Source–Detector Separation Distances

The current findings replicated the age-independent characteristics of changes in S-D channel DOT sensitivity profiles as a function of source–detector separation distance. The S-D channel DOT sensitivity profiles depict S-D channel DOT fluence as a function of sampling depth in the fluence distribution. The contribution of this study was to implement existing DOT sensitivity computations to age-specific realistic FEM head models ranging from 2 to 34 years with narrow age bands. Across the extended age span, the peak value of the S-D channel DOT fluence was greater at shorter separation distances but the S-D channel fluence value dissipated more rapidly as photons traveled through the head tissues. The peak value was reduced at larger separation distances but the fluence value showed less rapid decay with increased sampling depth. The between-channel variance of the S-D channel DOT sensitivity profiles was presented as the standard error of the mean fluence value as a function of sampling depth. All age groups displayed increased between-channel variance with increased sampling depth across separation distances. Lastly, we characterized the shape of the sensitivity profile by measuring the point in the fluence distribution where the fluence value first declined below half of the maximum value (i.e., HWHM location^10^^,^^48^). The HWHM location increased with source–detector separation distance for all age groups. The increase indicates that there is a gain in sensitivity to deeper cortical tissues with increased separation distances at the expense of decreased fluence strength. The present findings underscored the robust characteristics of the DOT sensitivity profiles found in existing studies using infant,^9^^,^^10^ child,^25^ and adult head models.^4^^,^^16^^,^^20^^,^^21^ Together, evidence shows that there is a trade-off between the gain in sensitivity to deeper cortical structures and loss in signal strength and spatial resolution.

The S-D channel DOT sensitivity displayed consistent patterns of age-related differences across source-detector separation distances. Young children (2 to 6 years) displayed distinctive sensitivity profiles compared to the older age groups, and the difference was more discernible at larger separation distances (e.g., 50 mm). The fluence values had lower peaks but declined less rapidly with increasing sampling depth across separation distances in the early childhood groups. The early childhood and late childhood groups (7 to 11 years) had shallower HWHM locations than the adolescence and adult groups across all separation distances. This indicates that the same fluence level was carried into deeper brain tissues in adolescents and adults. This finding is consistent with the differences in HWHM locations among age 2, 4, 12, and 20 to 24 years found in Ref. 10. Existing evidence also showed that the distance between scalp channel location and the maximum S-D channel DOT fluence in the cortex increased linearly from age 2 to 20 to 24 years.^18^ An important additional observation to Fu and Richards’s finding^10^ was that the between-age-group (individual age group as a categorical variable) and between-individual (age as a continuous variable) differences in sensitivity profiles increased with larger separation distances for children (2 to 11 years). The between-subject variations were more stable across separation distances in adolescents and adults. The between-subjects difference in the level of between-channel variance of S-D channel DOT fluence was also greater in young children than older individuals across separation distances.

The patterns of age-related differences in S-D channel DOT sensitivity profiles can be attributed to the developmental changes in head and brain structures. There is rapid growth in the whole head volume from toddlerhood to childhood, including skull thickness,^49^^,^^50^ CSF, GM, and WM volume.^12^^,^^24^^,^^51^ The developmental changes in brain volume (declining trend) decelerate from early adulthood and beyond.^24^^,^^52^ There are also between-individual differences in age-related changes of cortical structures during the first 6 years of life.^53^ The neurotypical age-related changes^24^^,^^52^ and individual differences in brain structural development may explain the current findings that young children (2 to 6 years) displayed distinctive sensitivity profiles compared to adolescents and adults, and young children showed greater between-subjects differences in the shape of sensitivity profiles and between-channel variations in sensitivity profiles. In addition, global cortical development is compounded by regional variations in structural changes.^51^ For example, cortical thickness follows a posterior-to-anterior maturational gradient.^54^ Young children (2 to 6 years) have smaller head sizes. Thus, larger source–detector separation may sample a larger variety of brain regions than older individuals with larger head sizes. This may have magnified the regional variations in structural differences and resulted in the observed greater between-channel variance of S-D channel DOT fluence in young children. Future research is needed to examine how within-person developmental changes and between-person variations in these changes are related to patterns of developmental changes in S-D channel DOT sensitivity profiles.

### 4.2 Applications of Age-Specific DOT Sensitivity Profiles

Our findings underscored the importance of using age-appropriate head models to make anatomical interpretations of NIRS/fNIRS data, when the participants’ own MRIs are not available. Accurate localizations of NIRS/fNIRS activities at the scalp channel into specific brain regions requires forward models that accurately describe the DOT sensitivity profiles. Realistic head models that account for age-related structural changes are optimal for accurate estimations of DOT sensitivity profiles that can guide channel placement on the scalp and DOT image reconstruction.^8^^,^^10^ Below, we further discuss the use of age-specific DOT sensitivity profiles in optimizing scalp channel placement and DOT image reconstruction.

The age-related differences in DOT sensitivity profiles underscore the importance of making age-specific decisions on the appropriate source–detector distances for the age group of interest. NIRS studies conventionally use 20 to 30 mm source–detector distances for infants and toddlers^1^^,^^55^ and 30 to 35 mm for children^56^^,^^57^ and adults.^58^ However, it is particularly important to make age-specific decisions on source–detector separation distances for children (2 to 11 years) considering the larger between-subject variations in S-D channel DOT sensitivity profiles among these age groups compared to adolescent and adult age groups. Across all separation distances, the fluence strength in the young children was lower than in older individuals as photons traveled to between about 15 to 45 mm into the head tissues. Young children also displayed lower peak fluence values, and the difference with older individuals enhanced at larger source–detector separation distances. It should also be noted that young children (e.g., 2 and 4 years) had close scalp-to-cortex distances averaged across scalp locations compared to adolescents (e.g., 12 years) and adults (e.g., 20 to 24 years).^18^ This means that DOT sensitivity to the cortex might be compatible among young children, adolescents, and adults at 20 to 30 mm separations conventionally used. Increasing separation distances beyond 30 mm for young children should be used with caution. Larger separation distances may cause reduction in signal strength, greater between-subject variations in DOT sensitivity, and greater between-channel variability in DOT sensitivity as photons travels deeper into the cortex.

Estimations of DOT sensitivity profiles by separation distances across development can be used to inform age-specific designs of channel/optode placement. Several studies have used the S-D channel DOT fluence measure to estimate the mapping between scalp channel locations and the underlying regions of interest.^19^^,^^59^^–^^63^ The devfOLD toolbox^19^ was extended from the fOLD toolbox^63^ using age-appropriate head models to estimate channel-to-ROI specificity for individual infant (2 weeks to 2 years with narrow age intervals), child (4 and 12 years), and adult (20 to 24 years) age groups. The devfOLD toolbox demonstrated that the between-age-group consistency or difference in channel-to-ROI mapping is driven by the targeted ROI and the desired specificity level, calculated as the percentage of channel sensitivity to the ROI out of the sensitivity to the brain. Higher sensitivity values could help to achieve between-age-group consistency in the channel configuration. The current findings illustrated that the between-age-group and between-subject variations in DOT sensitivity profiles were gradually reduced till adulthood. A good practice would be to use the averaged specificity values estimated using individual age-appropriate head models for designing channel/optode placement.^19^ The age-group averaged estimates can minimize errors caused by individual anatomical differences.^64^ The use of adult but age-unmatched head models for determining channel/optode placement for adult participants might be less problematic than using adult head models for developmental samples. However, future studies are needed to examine changes in DOT sensitivity profiles across larger age spans across early, middle, and late adulthood.

Future age-specific toolboxes for designing channel/optode placement need to include source–detector separation distance as an optimization parameter. The devfOLD toolbox^19^ has a constrained solution space that includes only 130 channels with predefined source and detector optodes on neighboring 10-10 positions. For example, the average separation distance across the 130 channels was 27.9 mm (SD=4.1, minimum=20.2  mm, maximum=37.6  mm) for the 2-year-olds, 28.4 mm (SD=4.3, minimum=20.7  mm, maximum=42.5  mm) for the 7-year-olds, 28.9 mm (SD=4.2, minimum=21.8  mm, maximum=45.1  mm) for the 12-year-olds, and 29.9 mm (SD=4.0, minimum=22.5  mm, maximum=42.9  mm) for the 20- to 24-year-olds. Thus, the toolbox-selected channels might not have the optimal source–detector separation distances that maximize the DOT sensitivity to the user-specific ROI and age group.^19^ The Array Designer toolbox^59^ decides channel positions based on user-specified ROI and separation distance range. However, customizations are required to incorporate age-specific optimizations. This study estimated S-D channel DOT sensitivity for a pool of 63,903 source–detector combinations with separation distances ranging from 10 to 60 mm. The estimations can be incorporated into future toolboxes that help to determine the optimal scalp channel locations and separation distances based on user-specified age group and ROI.

The present findings can inform the use of substitute head models for DOT image reconstruction in the absence of participants’ own MRIs. The age-related differences in DOT sensitivity profiles highlight the importance of using age-appropriate head models for DOT image reconstruction. The estimated DOT sensitivity profiles can be recorded into a forward matrix. This is then used for solving the inverse model that localizes the recorded hemoglobin concentration changes into specific cortical ROIs.^6^^,^^7^^,^^65^^,^^66^ Contemporary toolboxes for image reconstruction (AtlasViewer^67^ and TOAST++^68^) allow researchers to use an adult average template as the default option or user-specified an individual head model^66^^,^^69^ or an average template. It can be inferred from the present findings that the use of age-inappropriate average templates that have inaccurate representations of the anatomical geometry of the head and brain would produce erroneous forward and inverse models. Existing stand-alone fNIRS studies with developmental samples have used age-appropriate average templates for image reconstruction (examples Refs. 70 and 71). One limitation of age-appropriate average templates is that they may not account for individual anatomical differences.^66^ As discussed above, it can be beneficial to use an age-matched and head-size matched “close” substitute head model for solving the forward and the inverse models with enhanced accuracy when the participant’s own MRI is not available. The individual-based “close” head models have been used in existing studies that measured channel-to-ROI mapping by estimating S-D channel DOT profiles in developmental samples who may show larger individual differences in DOT sensitivity profiles compared to adults.^60^^–^^62^ Future studies are needed to systematically compare DOT image reconstruction results obtained from participant-specific head models, “close” head models, age-appropriate average templates, and age-inappropriate average templates.

### 4.3 Limitations

There is no consensus on the optical properties in the literature.^10^^,^^63^ The values also vary across adult studies.^4^^,^^5^^,^^17^^,^^22^^,^^63^^,^^72^ Our input values for optical properties were based on values used in Ref. 73, which used values specified in several prior studies.^22^^,^^74^^,^^75^ This study conducted fine-grained segmentation of tissues layers. However, there is limited reference for appropriate optical property values for the different tissue types. We did not differentiate optical property values for some extracerebral tissue layers including the skin, dura, muscle, and nasal cavity, as well as the refractive index across tissue types. Some studies have used the same refractive index across tissue segments as the present study.^17^^,^^63^^,^^73^^,^^74^^,^^76^ However, it is likely that different refractive indices should be applied to extracerebral, CSF, and brain tissues.^25^^,^^77^ We also did not apply age-specific optical properties. We acknowledge that determining the optimal optical properties for different tissue layers is challenging. The established values for various tissue types have largely been obtained *in vitro* using different instrument and measurement methods. The optical properties *in vivo* can vary from person to person, age group to age group, and time to time.^78^ Future studies are needed to examine the impact of specifying age-appropriate optical property values on the patterns of age-related differences in DOT sensitivity profiles across separation distances.

Moreover, this study did not compare S-D channel DOT sensitivity profiles among different wavelengths or tissue layers. Studies have simulated light propagations through head tissues using multiple wavelengths commonly used in NIR devices (e.g., from 690 to 850 nm) in children^25^ and adults.^4^^,^^77^ There are wavelength-related differences in light deposition on the brain.^77^ However, we expect that the differences in DOT sensitivity profiles would be subtle across the NIR wavelengths based on existing findings.^4^^,^^77^ It is also likely that the change of relative sensitivity as a function of separation distance displays different shapes for GM, WM, CSF, and extracerebral tissues.^4^^,^^9^^,^^16^^,^^20^ An extension of this study is to compare the relative DOT sensitivity to the GM versus extracerebral tissues (e.g., the scalp), separately by the target source–detector separation distances and age groups.^4^^,^^17^ The estimations could be used for making age-specific decisions on the short-separation channels that enable researchers to regress hemodynamic signals of the scalp from signals of standard separation channels.^17^

### 4.4 Conclusions

This study examined S-D channel DOT sensitivity profiles as a function of source–detector separation distances from age 2 years to 30 to 34 years with narrow age internals. We replicated existing evidence demonstrating that the sensitivity profiles change systematically as a function of source–detector separation distance for all age groups. There were also important age-related differences in the S-D channel DOT sensitivity profiles. Young children (2 to 6 years) displayed distinctive shape of sensitivity profiles, larger between-subject differences, and between-channel variations in sensitivity profiles compared to older individuals, and the differences were particularly discernible at larger separation distances. Our findings of age-related differences in S-D channel DOT sensitivity profiles have important implications in determining age-appropriate source–detector separation distances and placement that match sensitivity, signal strength, and penetration depth for different ages. Our findings have also highlighted that standalone DOT studies without MRI data collections should use age-appropriate realistic head models that can accurately describe age-related anatomical changes to estimate S-D channel DOT sensitivity profiles. Optimization of channel configurations and accurate anatomical interpretations of NIRS/fNIRS data are dependent on age-specific estimations of S-D channel DOT sensitivity profiles that account for the head and cortical changes throughout the lifespan.

## Code, Data, and Materials Availability

The age-specific average templates, including T1, DOT sensitivity average template can be accessed from the “Neurodevelopmental MRI Database.” The database is available online: http://jerlab.psych.sc.edu/NeurodevelopmentalMRIDatabase/ for information and https://www.nitrc.org/projects/neurodevdata/for access. Details for the online access are provided in Ref. 34. The codes that support the findings of this study are available from the corresponding author, XF, upon request.
