# Supplementary material for: Age-related changes in diffuse optical tomography sensitivity profiles from childhood to adulthood
Source: J Biomed Opt. 2022 Jul 9;27(8):083004. doi: 10.1117/1.JBO.27.8.083004 (PMC9270691; doi:10.1117/1.JBO.27.8.083004)
Supplement: Supplementary file 1 [file JBO_027_083004_SD001.pdf]

Supplemental Information

MRI Segmentation

Each head MRI volume was segmented into 9 media types: gray matter (GM), white matter (WM), cerebrospinal fluid (CSF), other brain matter, skin, skull, air, eyes, and other inside skull material. The FSL FAST procedure [1] was used to segment the T1-weighted images into GM, WM, or other matter (OM). The CSF was identified in the T2W images using a threshold procedure. The CSF was removed from the materials from the FAST procedure, with the remainder defined as GM, WM, or other inside skull material. The BETSURF procedure [2,3] was used with the extracted brain, T1W and T2W volumes, to identify skull and scalp regions. The nasal cavity and eyes were identified manually using MRIcron [4,5]. Finally, any other matter inside the head volume not defined as above was defined as "other inside skull material". This generally was in the region of the neck and consisted primarily of muscle and secondarily of spinal bone.

10-10 Electrodes Placement

The 81 10-10 electrode locations were constructed based on the "unambiguously illustrated 10-10 system [6]. The virtual optodes placements at 10-10 electrode positions are displayed in Main Text Figure We divided the electrode positions to six groups for visualization purposes. The Cz was located at the intersection of the front-to-back central curve (Nz to Iz) and the left-to-right central curve (LPA to RPA). The "z" electrodes were placed at the 10% intervals on the Nz-Cz-Iz central curve. The LPA-RPA central curve was divided in 10% increments to set electrodes T7 to T8. From Nz to LPA to Iz, the N1, I1, and the "9" electrodes (e.g. AF9 and PO9) were identified in 10% intervals, and likewise for the N2, I2, and the "10" electrodes (e.g. AF10 and PO10) set on the right hemisphere. The curve Fpz to T7 to Oz was divided in 10%

increments to generate Fp1, O1, and the “7” electrodes, and the same method was applied to identify Fp2, O2, and the “8” electrodes on the right hemisphere. Lastly, the “z”, “7” and “8” locations were used to define “1” and “2”, ‘3” and “4”, and “5 and “6” electrode locations.

Supplemental Table 1. The mean minimum and maximum source-detector separation distances for each target separation distance for each age group. The minimum and maximum separation distances were set to ensure about 500 channels are being samples for each target separation.

Age Group	Separation Distance (mm)	Minimum Distance (mm)	Maximum Distance (mm)
2-0Years	10	5.68	14.32
2-0Years	15	13.11	16.89
2-0Years	20	18.03	21.97
2-0Years	25	23.25	26.75
2-0Years	30	28.84	31.16
2-0Years	35	33.83	36.17
2-0Years	40	38.87	41.13
2-0Years	45	44.1	45.9
2-0Years	50	49.13	50.87
2-0Years	55	54.12	55.88
2-0Years	60	59.23	60.77
3-0Years	10	5.67	14.33
3-0Years	15	13.11	16.89
3-0Years	20	18.03	21.97
3-0Years	25	23.24	26.76
3-0Years	30	28.85	31.15
3-0Years	35	33.8	36.2
3-0Years	40	38.87	41.13
3-0Years	45	44.09	45.91
3-0Years	50	49.11	50.89
3-0Years	55	54.12	55.88
3-0Years	60	59.23	60.77
4-0Years	10	6	14
4-0Years	15	13.5	16.5
4-0Years	20	18.3	21.7
4-0Years	25	23.5	26.5
4-0Years	30	29	31
4-0Years	35	34	36
4-0Years	40	39	41
4-0Years	45	44.3	45.7
4-0Years	50	49.3	50.7
4-0Years	55	54.3	55.7
4-0Years	60	59.3	60.7
5-0Years	10	4.85	15.15

5-0Years	15	12.85	17.15
5-0Years	20	17.84	22.16
5-0Years	25	23.08	26.92
5-0Years	30	28.55	31.45
5-0Years	35	33.71	36.29
5-0Years	40	38.72	41.28
5-0Years	45	43.93	46.07
5-0Years	50	49	51
5-0Years	55	54.03	55.97
5-0Years	60	59.11	60.89
6-0Years	10	5.58	14.42
6-0Years	15	13.2	16.8
6-0Years	20	17.88	22.12
6-0Years	25	23.19	26.81
6-0Years	30	28.78	31.22
6-0Years	35	33.76	36.24
6-0Years	40	38.81	41.19
6-0Years	45	44.03	45.98
6-0Years	50	49.14	50.86
6-0Years	55	54.09	55.91
6-0Years	60	59.17	60.83
7-0Years	10	5.88	14.12
7-0Years	15	13.12	16.88
7-0Years	20	17.99	22.01
7-0Years	25	23.28	26.72
7-0Years	30	28.73	31.27
7-0Years	35	33.82	36.18
7-0Years	40	38.84	41.16
7-0Years	45	44	46
7-0Years	50	49.1	50.9
7-0Years	55	54.09	55.91
7-0Years	60	59.15	60.85
8-0Years	10	5.29	14.71
8-0Years	15	13.21	16.79
8-0Years	20	17.72	22.28
8-0Years	25	23.16	26.84
8-0Years	30	28.71	31.29
8-0Years	35	33.72	36.28
8-0Years	40	38.77	41.23
8-0Years	45	43.99	46.01
8-0Years	50	49.08	50.92
8-0Years	55	54.07	55.93
8-0Years	60	59.12	60.88
9-0Years	10	5.15	14.85

9-0Years	15	13.11	16.89
9-0Years	20	17.84	22.16
9-0Years	25	23.1	26.9
9-0Years	30	28.68	31.32
9-0Years	35	33.79	36.21
9-0Years	40	38.75	41.25
9-0Years	45	43.96	46.04
9-0Years	50	49.06	50.94
9-0Years	55	54.1	55.9
9-0Years	60	59.1	60.9
10-0Years	10	4.94	15.06
10-0Years	15	13.14	16.86
10-0Years	20	17.66	22.34
10-0Years	25	23.02	26.98
10-0Years	30	28.66	31.34
10-0Years	35	33.77	36.23
10-0Years	40	38.66	41.34
10-0Years	45	43.98	46.02
10-0Years	50	49.02	50.98
10-0Years	55	54.08	55.92
10-0Years	60	59.07	60.93
11-0Years	10	5.75	14.26
11-0Years	15	12.99	17.02
11-0Years	20	17.59	22.41
11-0Years	25	22.78	27.22
11-0Years	30	28.65	31.36
11-0Years	35	33.65	36.36
11-0Years	40	38.64	41.37
11-0Years	45	43.89	46.12
11-0Years	50	49	51
11-0Years	55	53.98	56.02
11-0Years	60	59.1	60.9
12-0Years	10	5.5	14.5
12-0Years	15	13.5	16.5
12-0Years	20	18	22
12-0Years	25	23.4	26.6
12-0Years	30	28.8	31.2
12-0Years	35	34	36
12-0Years	40	38.9	41.1
12-0Years	45	44.2	45.8
12-0Years	50	49.2	50.8
12-0Years	55	54.2	55.8
12-0Years	60	59.2	60.8
12-5Years	10	4.85	15.15

12-5Years	15	13.18	16.82
12-5Years	20	17.64	22.36
12-5Years	25	22.98	27.02
12-5Years	30	28.62	31.38
12-5Years	35	33.76	36.24
12-5Years	40	38.65	41.35
12-5Years	45	43.93	46.07
12-5Years	50	49.05	50.95
12-5Years	55	54.03	55.97
12-5Years	60	59.09	60.91
13-0Years	10	4.91	15.09
13-0Years	15	12.69	17.31
13-0Years	20	16.88	23.12
13-0Years	25	22	28
13-0Years	30	27.46	32.54
13-0Years	35	32.26	37.74
13-0Years	40	37	43
13-0Years	45	42.08	47.92
13-0Years	50	46.9	53.1
13-0Years	55	51.71	58.29
13-0Years	60	56.54	63.46
13-5Years	10	5.1	14.9
13-5Years	15	13.23	16.77
13-5Years	20	17.67	22.34
13-5Years	25	23.03	26.97
13-5Years	30	28.68	31.32
13-5Years	35	33.72	36.28
13-5Years	40	38.69	41.31
13-5Years	45	43.99	46.01
13-5Years	50	49.03	50.97
13-5Years	55	54.07	55.93
13-5Years	60	59.11	60.89
14-0Years	10	4.92	15.08
14-0Years	15	13.13	16.87
14-0Years	20	17.66	22.34
14-0Years	25	22.95	27.05
14-0Years	30	28.6	31.4
14-0Years	35	33.74	36.26
14-0Years	40	38.65	41.35
14-0Years	45	43.92	46.08
14-0Years	50	49.01	50.99
14-0Years	55	54.04	55.96
14-0Years	60	59.09	60.91
15-0Years	10	4.87	15.13

15-0Years	15	13.03	16.97
15-0Years	20	17.69	22.31
15-0Years	25	22.95	27.05
15-0Years	30	28.58	31.42
15-0Years	35	33.76	36.24
15-0Years	40	38.64	41.36
15-0Years	45	43.87	46.13
15-0Years	50	49.02	50.98
15-0Years	55	54.02	55.98
15-0Years	60	59.09	60.91
16-0Years	10	4.53	15.47
16-0Years	15	13.04	16.96
16-0Years	20	17.67	22.33
16-0Years	25	22.91	27.09
16-0Years	30	28.51	31.49
16-0Years	35	33.76	36.24
16-0Years	40	38.61	41.39
16-0Years	45	43.87	46.13
16-0Years	50	49.02	50.98
16-0Years	55	54.02	55.98
16-0Years	60	59.06	60.94
17-0Years	10	4.8	15.2
17-0Years	15	13.11	16.89
17-0Years	20	17.75	22.25
17-0Years	25	22.87	27.13
17-0Years	30	28.64	31.36
17-0Years	35	33.74	36.26
17-0Years	40	38.61	41.39
17-0Years	45	43.97	46.03
17-0Years	50	49.04	50.96
17-0Years	55	54.03	55.97
17-0Years	60	59.1	60.9
18-0Years	10	4.74	15.26
18-0Years	15	13.09	16.91
18-0Years	20	17.69	22.31
18-0Years	25	22.92	27.08
18-0Years	30	28.57	31.43
18-0Years	35	33.71	36.29
18-0Years	40	38.66	41.34
18-0Years	45	43.9	46.1
18-0Years	50	49	51
18-0Years	55	54.02	55.98
18-0Years	60	59.07	60.93
18-5Years	10	4.78	15.22

18-5Years	15	13.14	16.86
18-5Years	20	17.66	22.34
18-5Years	25	22.93	27.07
18-5Years	30	28.58	31.42
18-5Years	35	33.75	36.25
18-5Years	40	38.63	41.37
18-5Years	45	43.91	46.09
18-5Years	50	49.02	50.98
18-5Years	55	54.03	55.97
18-5Years	60	59.05	60.95
19-0Years	10	4.96	15.04
19-0Years	15	13.24	16.76
19-0Years	20	17.63	22.37
19-0Years	25	22.94	27.06
19-0Years	30	28.64	31.36
19-0Years	35	33.72	36.28
19-0Years	40	38.66	41.34
19-0Years	45	43.97	46.03
19-0Years	50	49.02	50.98
19-0Years	55	54.04	55.96
19-0Years	60	59.1	60.9
19-5Years	10	4.79	15.21
19-5Years	15	13.17	16.83
19-5Years	20	17.67	22.33
19-5Years	25	22.91	27.09
19-5Years	30	28.6	31.4
19-5Years	35	33.74	36.26
19-5Years	40	38.62	41.39
19-5Years	45	43.94	46.06
19-5Years	50	49.03	50.97
19-5Years	55	54.04	55.96
19-5Years	60	59.07	60.93
20-24Years	10	5.08	14.92
20-24Years	15	13.2	16.8
20-24Years	20	17.99	22.01
20-24Years	25	23.19	26.81
20-24Years	30	28.7	31.3
20-24Years	35	33.99	36.01
20-24Years	40	38.8	41.2
20-24Years	45	44	46
20-24Years	50	49.2	50.8
20-24Years	55	54.19	55.81
20-24Years	60	59.29	60.71
25-29Years	10	4.72	15.28

25-29Years	15	13.06	16.94
25-29Years	20	17.67	22.33
25-29Years	25	22.91	27.09
25-29Years	30	28.57	31.43
25-29Years	35	33.7	36.3
25-29Years	40	38.64	41.36
25-29Years	45	43.89	46.11
25-29Years	50	48.99	51.01
25-29Years	55	54.03	55.97
25-29Years	60	59.06	60.94
30-34Years	10	4.55	15.45
30-34Years	15	12.97	17.03
30-34Years	20	17.69	22.31
30-34Years	25	22.73	27.27
30-34Years	30	28.16	31.84
30-34Years	35	33.25	36.75
30-34Years	40	38.04	41.96
30-34Years	45	43.06	46.94
30-34Years	50	48.05	51.95
30-34Years	55	52.93	57.07
30-34Years	60	57.82	62.18

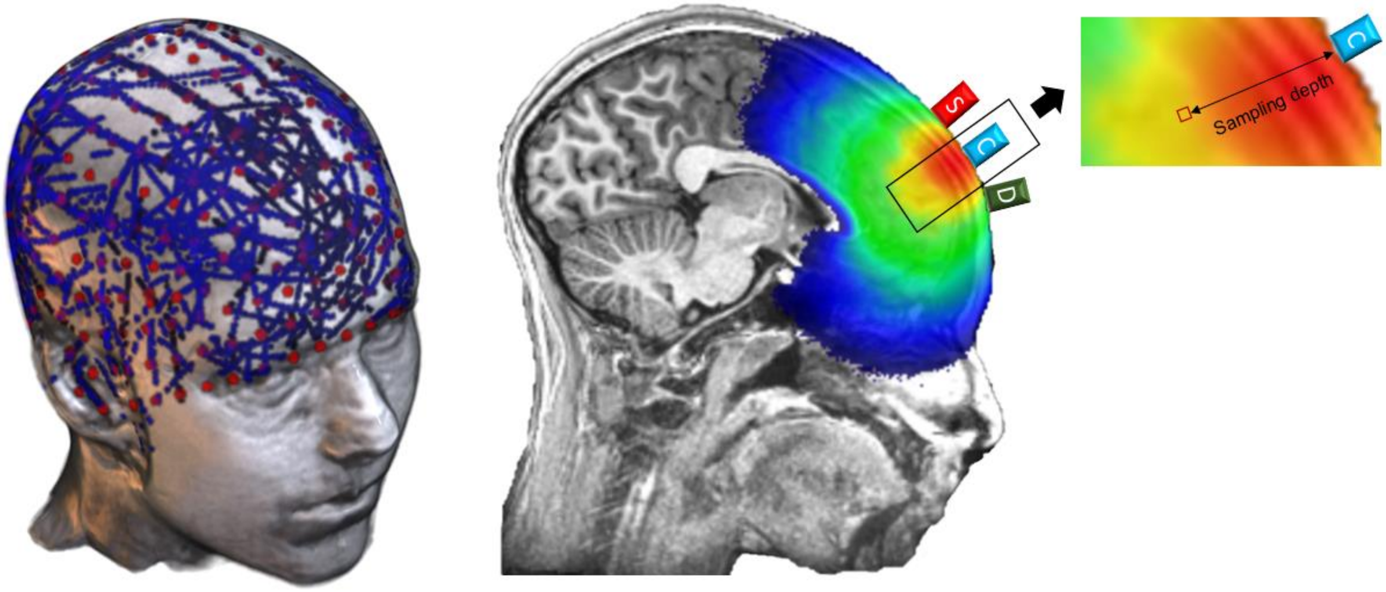

SI Fig 1. Illustrations of source-detector channels and sampling depth for computing Source-Detector DOT Sensitivity Profiles. The left panel present an example of selected channels for a 12-year-old participant. There was a total of 501 channels sampled at the minimum separation distance of 28.8 mm and maximum separation distance of 31.2 mm for the target separation distance of 30 mm. The right panel presents a conceptual illustration of the computation of sampling depth. It is the distance from the channel location to the voxel location of the Source-Detector Channel DOT fluence value inside the head model.

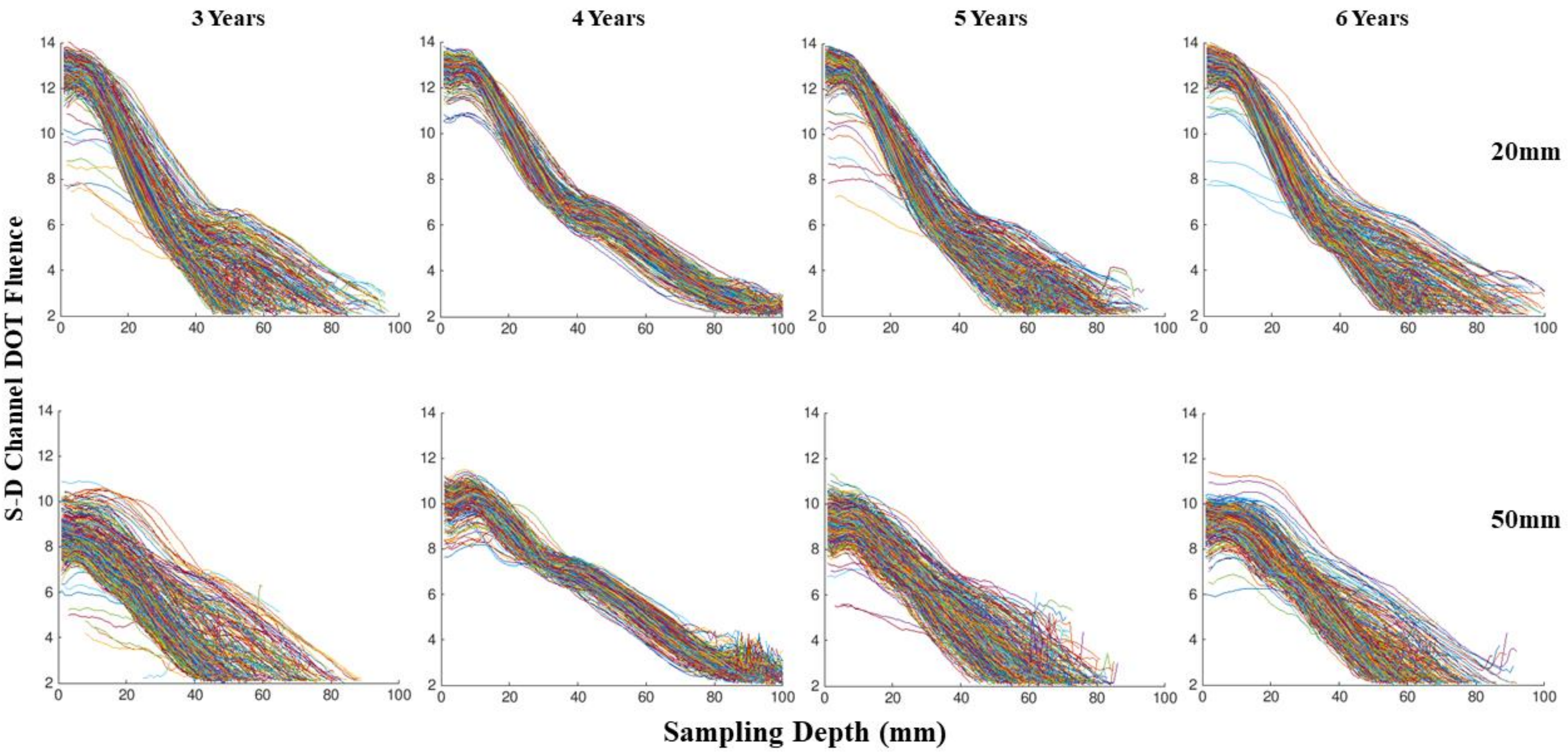
3 Years
4 Years
5 Years
6 Years
20mm
50mm
S-D Channel DOT Fluence
Sampling Depth (mm)

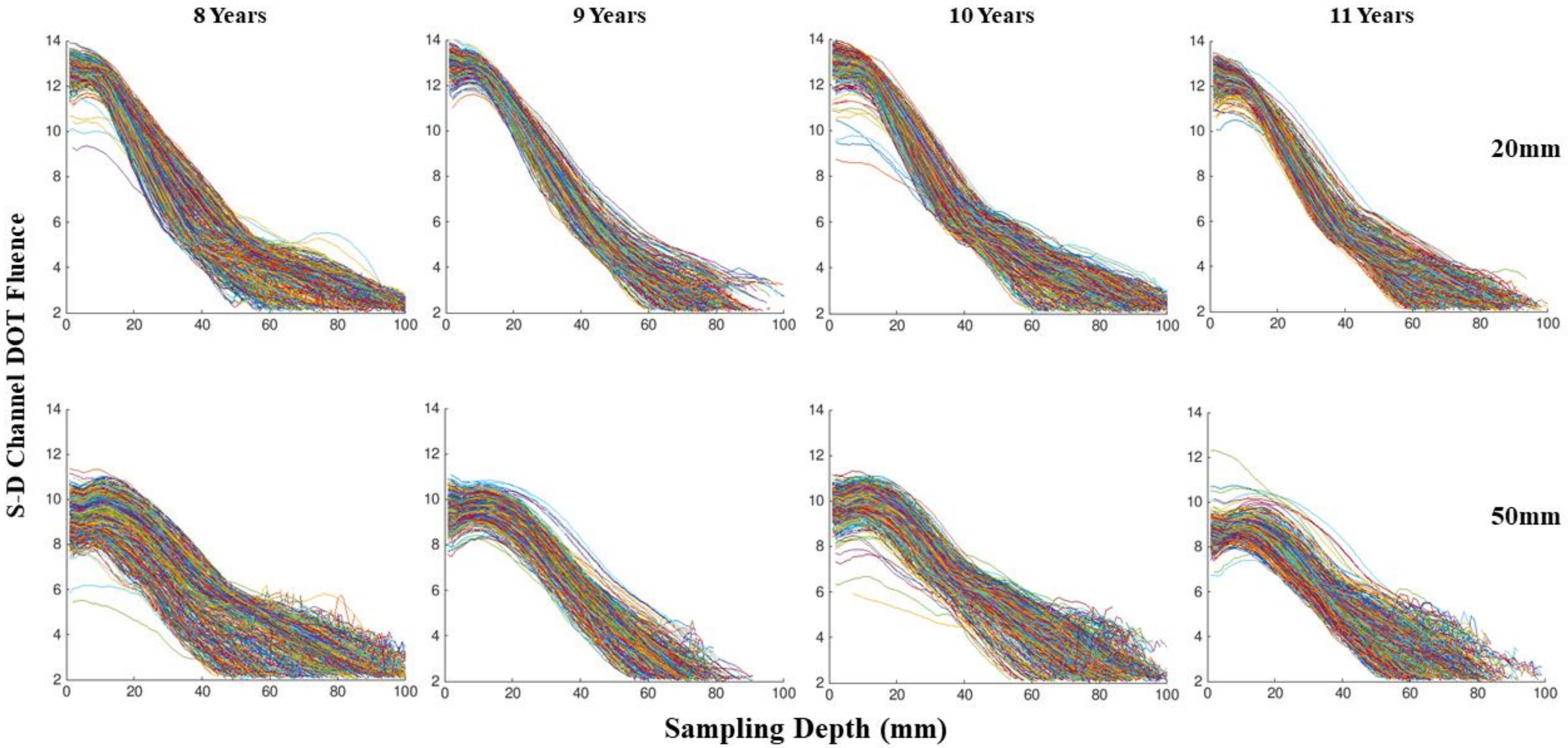
8 Years
9 Years
10 Years
11 Years
20mm
50mm
S-D Channel DOT Fluence
Sampling Depth (mm)

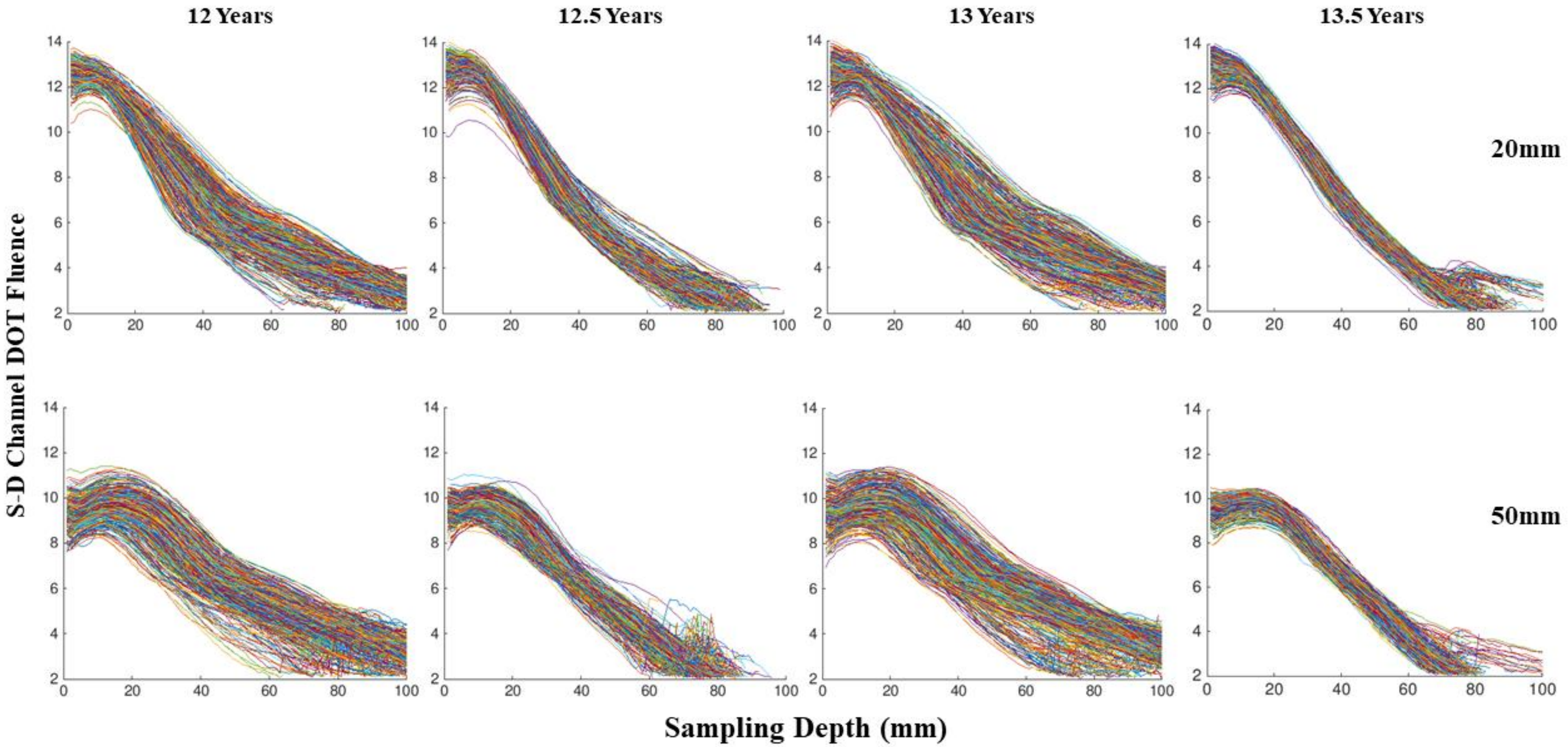
12 Years
12.5 Years
13 Years
13.5 Years
20mm
50mm
S-D Channel DOT Fluence
Sampling Depth (mm)

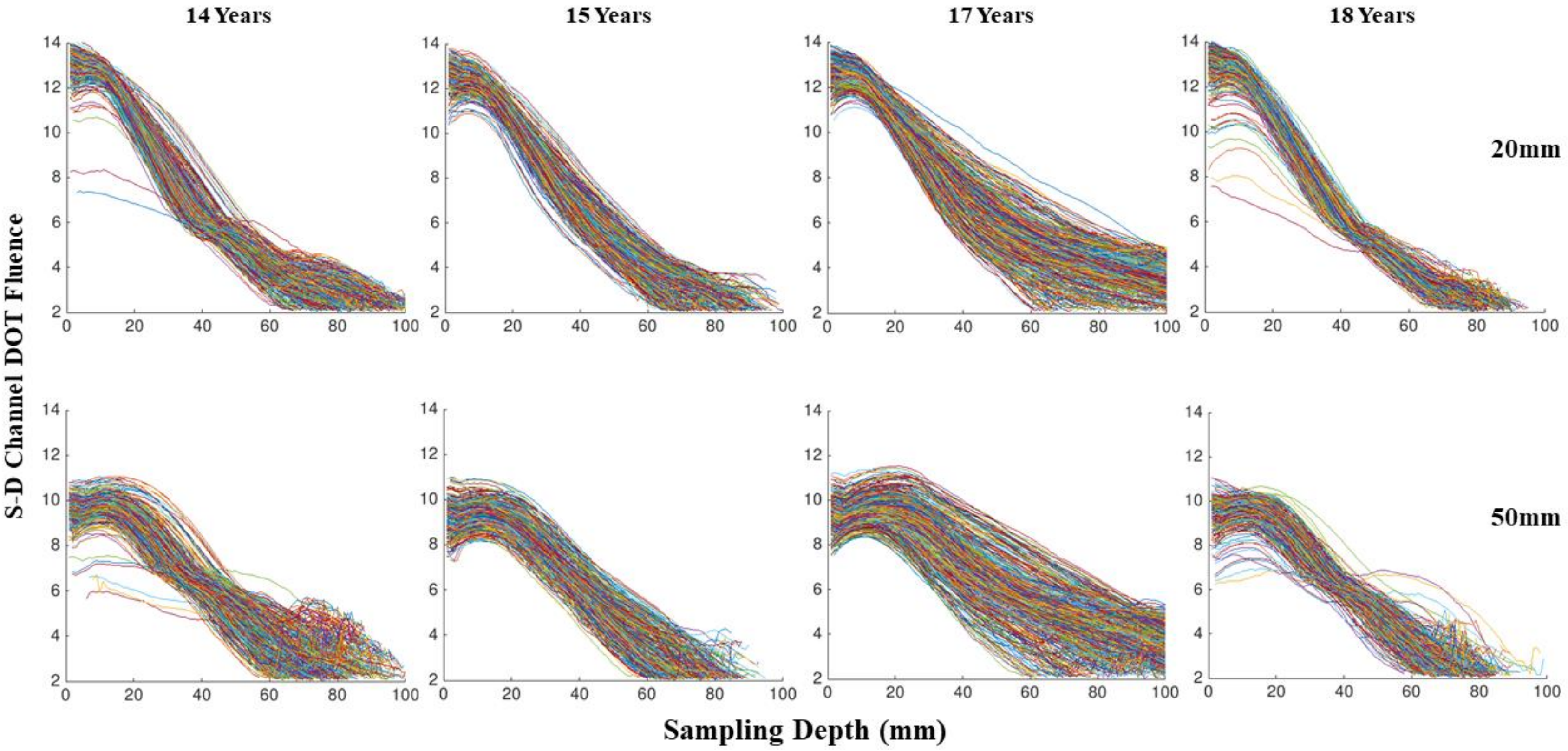
14 Years
15 Years
17 Years
18 Years
20mm
50mm
S-D Channel DOT Fluence
Sampling Depth (mm)

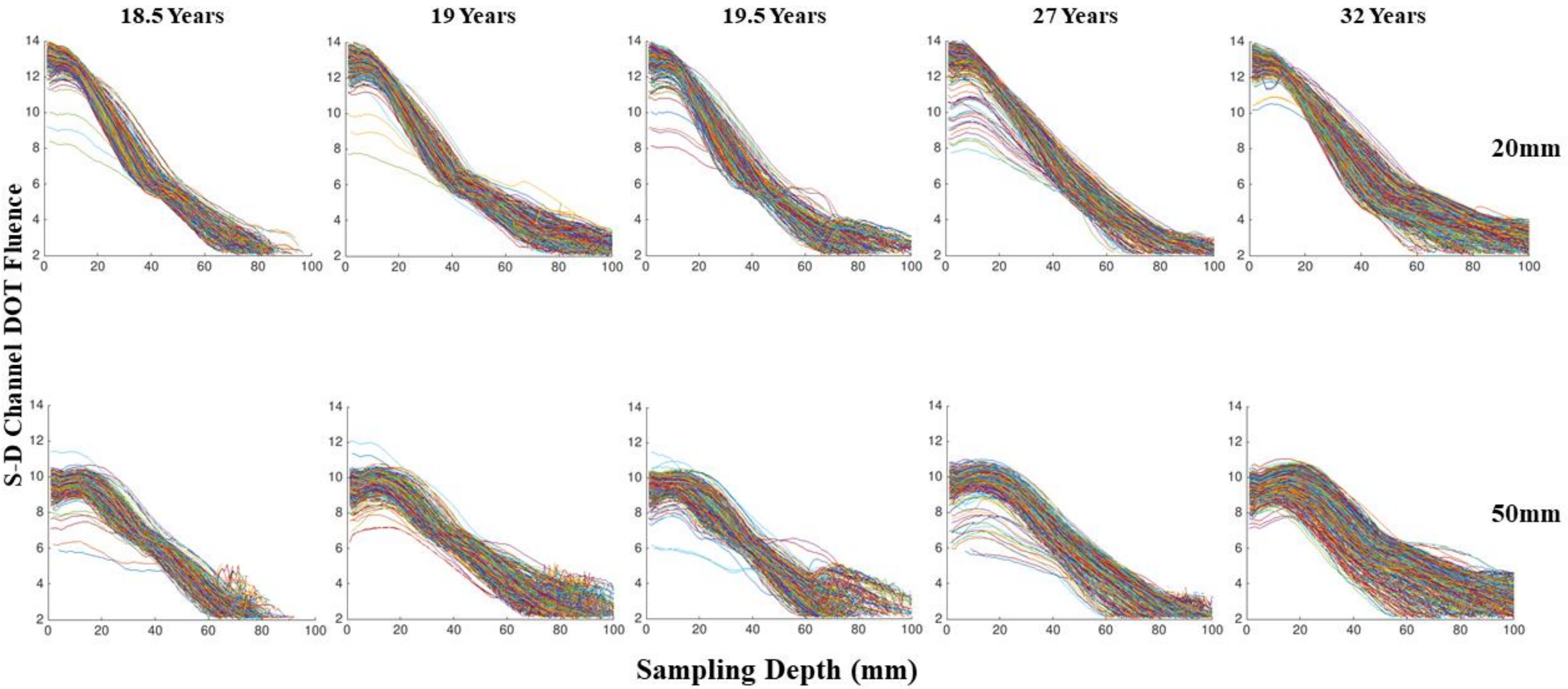

SI Fig 2. Source-Detector (S-D) Channel DOT fluence sensitivity profile by channels. One example individual was selected for each age group. Additional examples are provided in the Main Text. For each participant and each channel with the target separation distance, we computed the S-D Channel DOT fluence and the distance from the channel location to the voxel with the fluence estimation ("sampling depth"). The S-D Channel DOT fluence value was plotted as a function of the sampling depth separately for channels at the source-detector separation distances for 20 mm and 50 mm for everyone.

A.

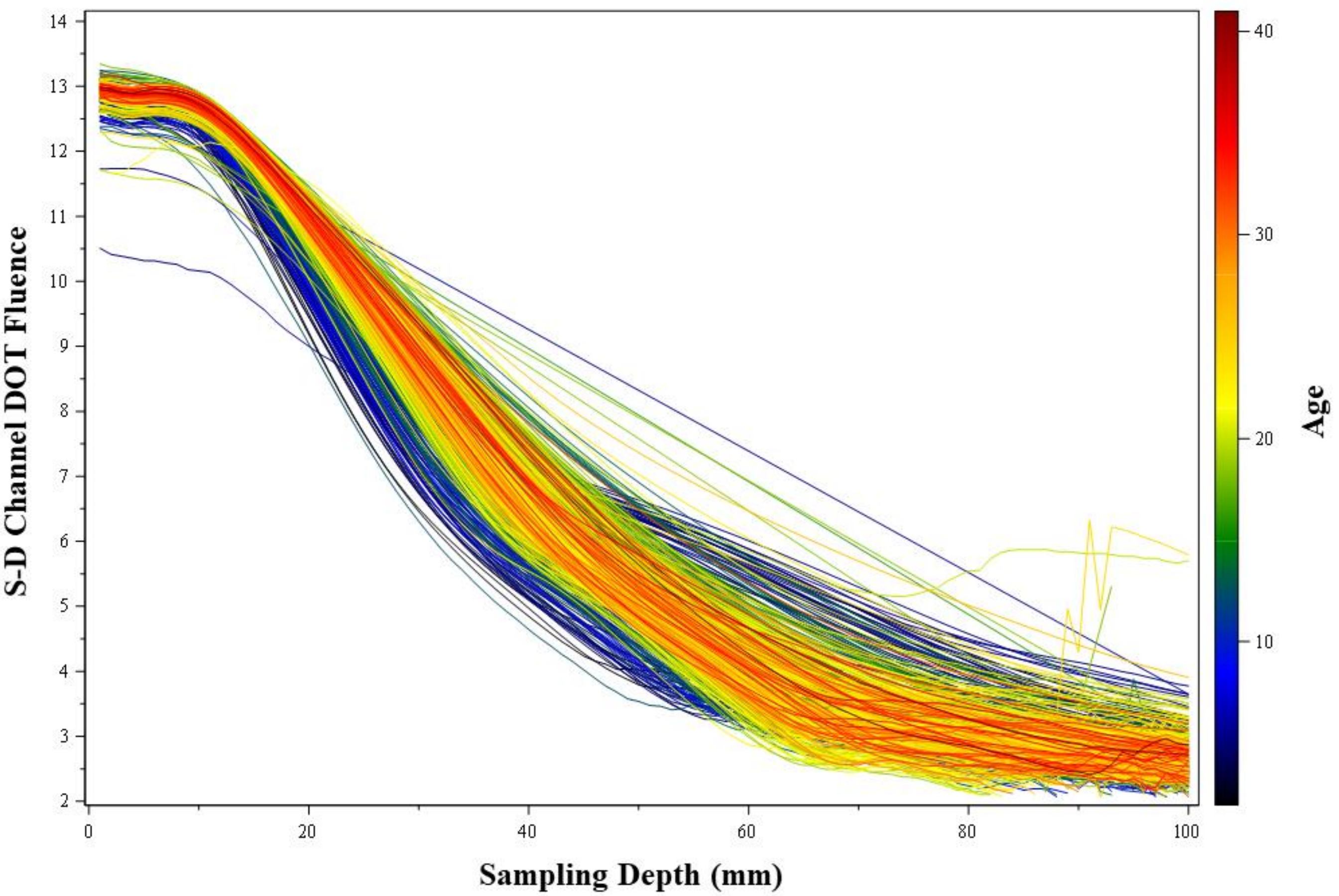

S-D Channel DOT Fluence
Sampling Depth (mm)
Age
14
13
12
11
10
9
8
7
6
5
4
3
2
0
20
40
60
80
100
40
30
20
10

B.

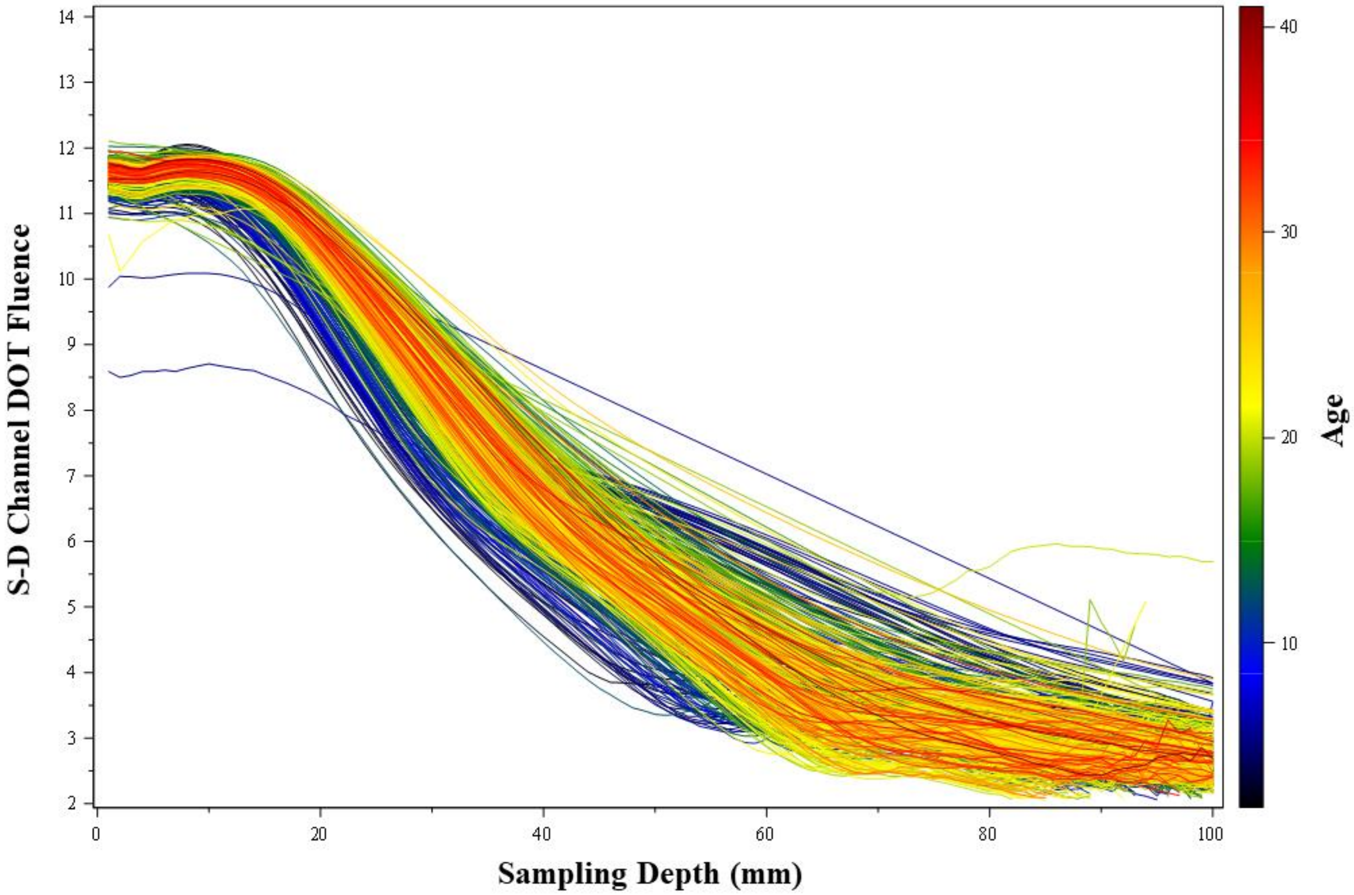
S-D Channel DOT Fluence
Sampling Depth (mm)
Age
14
13
12
11
10
9
8
7
6
5
4
3
2
0
20
40
60
80
100
40
30
20
10

C.

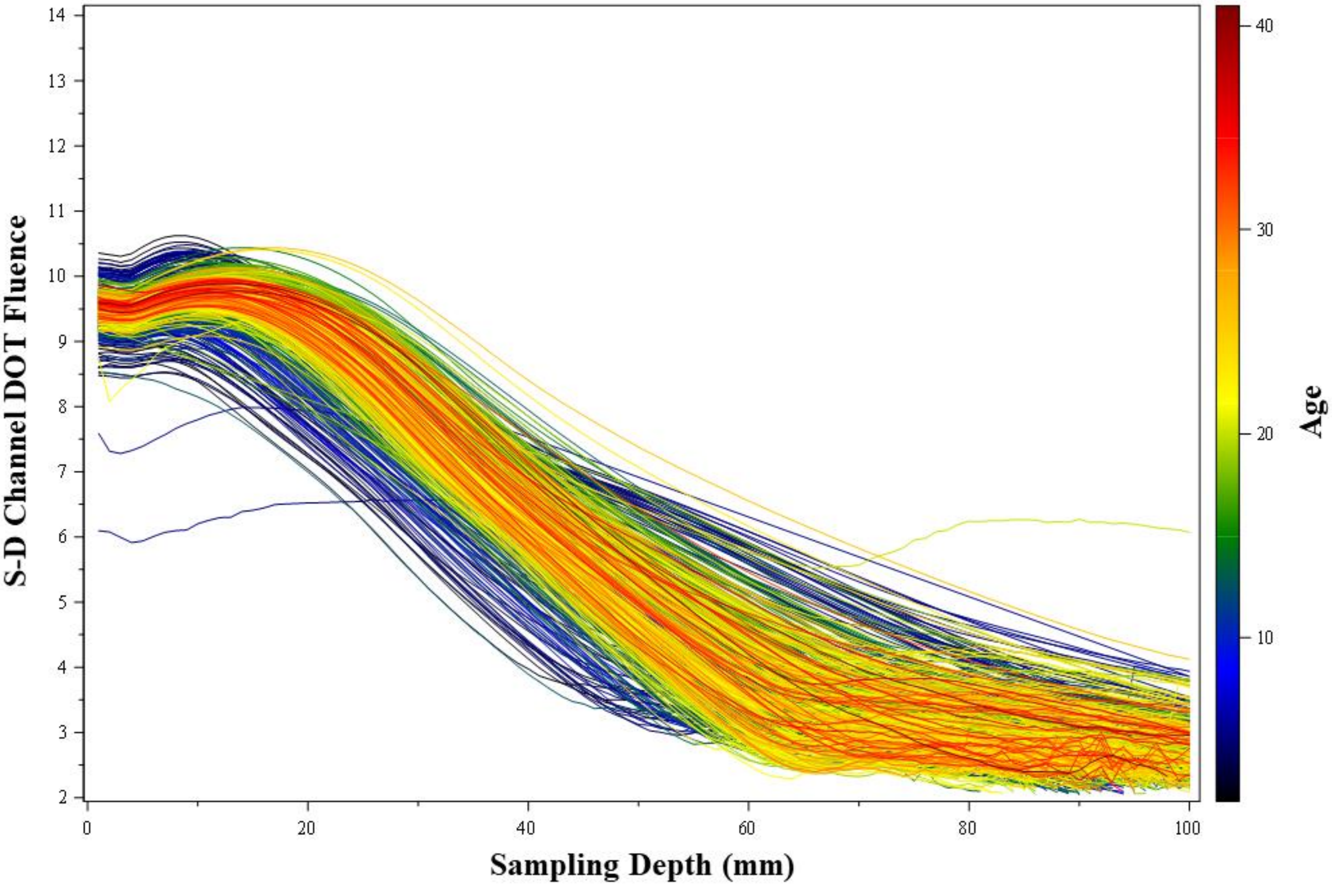

SI Fig 3. Source-Detector (S-D) Channel DOT fluence sensitivity profile by participants' ages. A. S-D Channel DOT fluence sensitivity profile at 20 mm source-detector separation. B. S-D Channel DOT fluence sensitivity profile at 30 mm source-detector separation. C. S-D Channel DOT fluence sensitivity profile at 50 mm source-detector separation.

A.

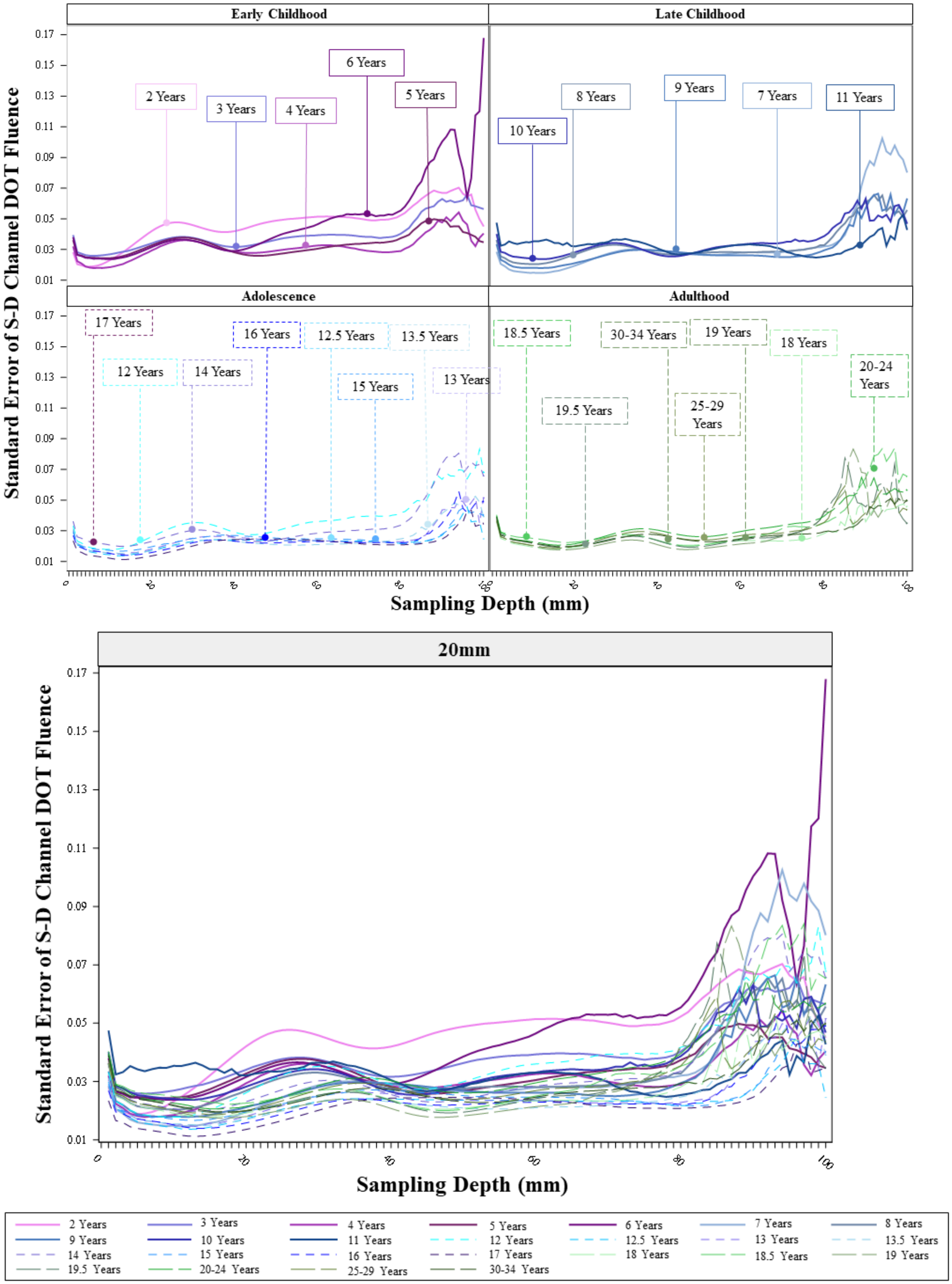
Early Childhood
Late Childhood
Adolescence
Adulthood
2 Years
3 Years
4 Years
5 Years
6 Years
10 Years
8 Years
9 Years
7 Years
11 Years
17 Years
12 Years
14 Years
16 Years
12.5 Years
13.5 Years
15 Years
13 Years
18.5 Years
19.5 Years
30-34 Years
25-29 Years
19 Years
18 Years
20-24 Years
Standard Error of S-D Channel DOT Fluence
Sampling Depth (mm)
20mm
0.01
0.03
0.05
0.07
0.09
0.11
0.13
0.15
0.17
0
20
40
60
80
100
2 Years
3 Years
4 Years
5 Years
6 Years
7 Years
8 Years
9 Years
10 Years
11 Years
12 Years
12.5 Years
13 Years
13.5 Years
14 Years
15 Years
16 Years
17 Years
18 Years
18.5 Years
19 Years
19.5 Years
20-24 Years
25-29 Years
30-34 Years

B.

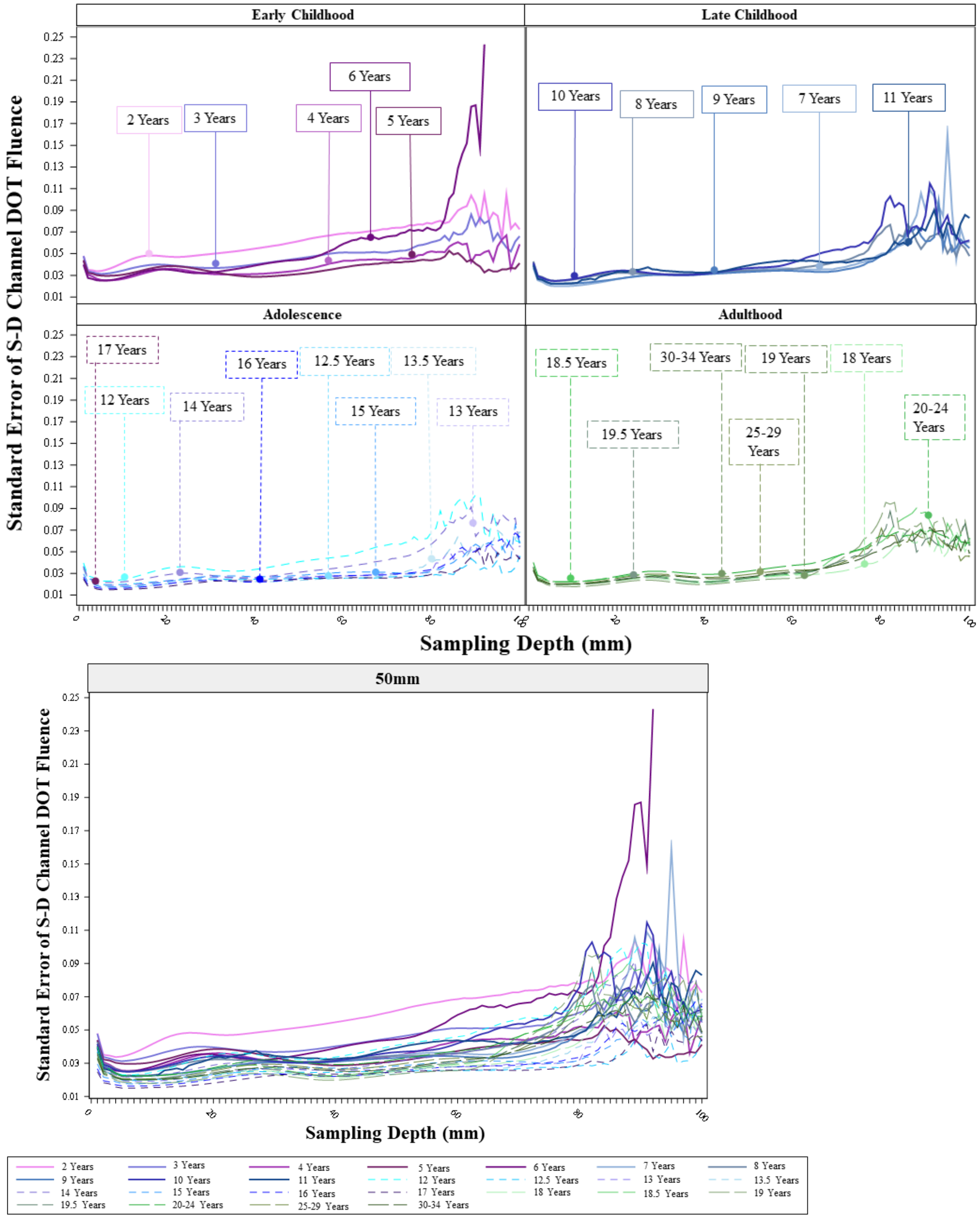

SI Fig 4. Standard error of the Source-Detector (S-D) Channel DOT fluence sensitivity profile by individual age groups. A. Standard error of the sensitivity profile at 20 mm source-detector separation distance. B. Standard error of the sensitivity profile at 50 mm separation distance. The upper panel presents the variance as a function of sampling depth by age groups in age bins (as separate panels). The lower panel plots the variance by age groups in the same panel.

A.

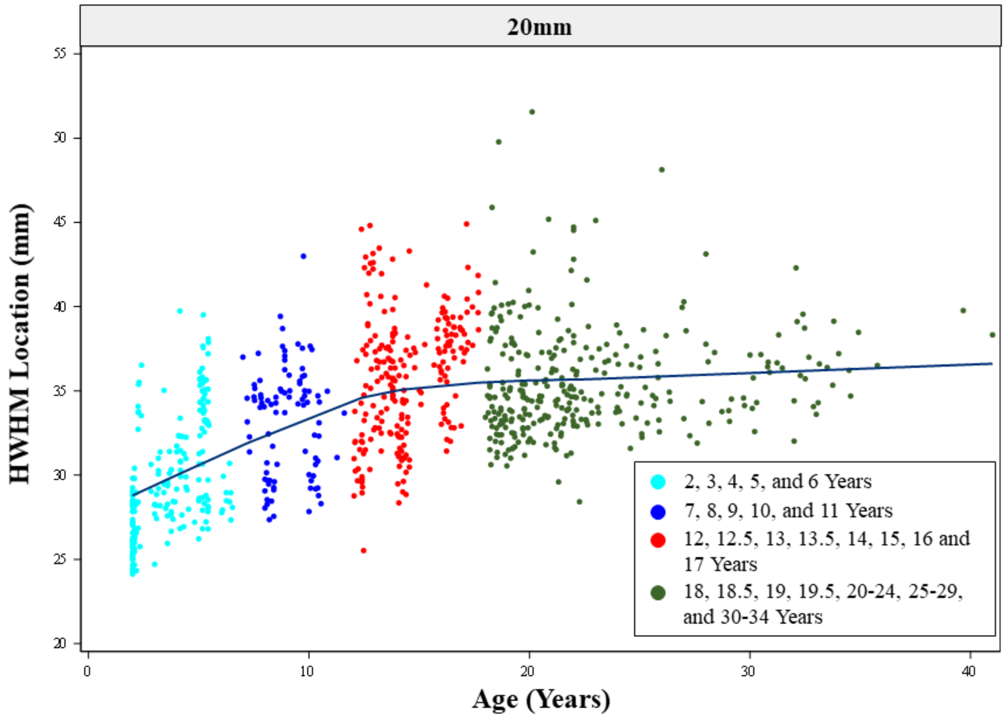

B.

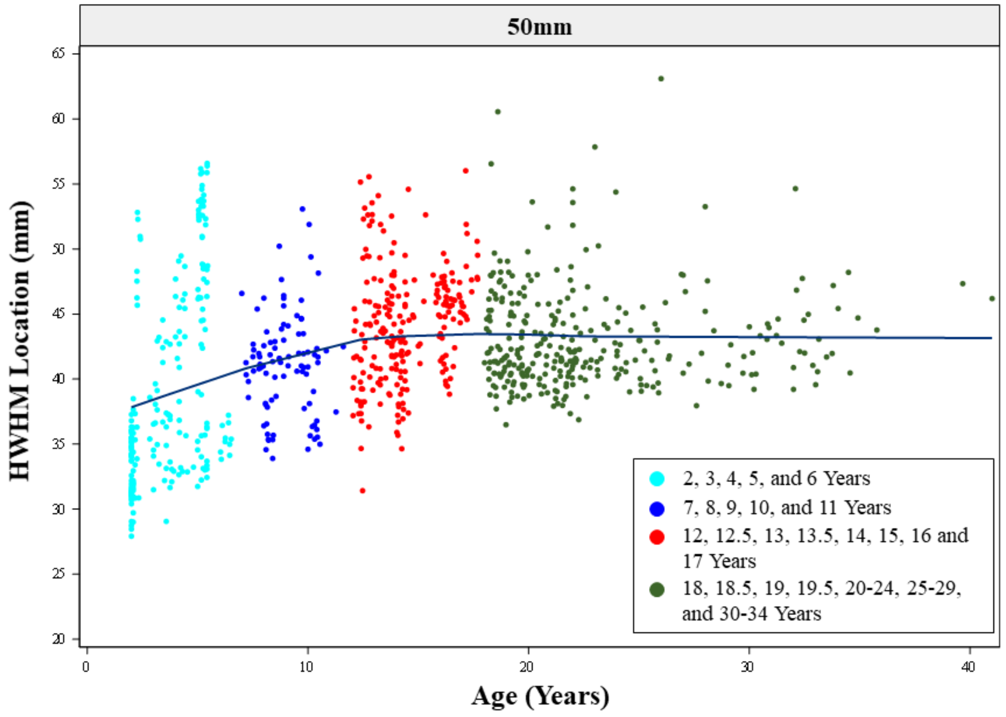

SI Fig 5. Half-width half-maximum (HWHM) locations of Source-Detector (S-D) Channel fluence as a function of age. The ages of individual participants were used as a continuous variable. the four age bins (early childhood, late childhood, adolescence, and adulthood) were color-coded. A. HWMH locations at 20mm source-detector separation. B. HWMH locations at 50mm source-detector separation.
